# How are social stressors at work related to well-being and health? A systematic review and meta-analysis

**DOI:** 10.1186/s12889-021-10894-7

**Published:** 2021-05-10

**Authors:** Christin Gerhardt, Norbert K. Semmer, Sabine Sauter, Alexandra Walker, Nathal de Wijn, Wolfgang Kälin, Maria U. Kottwitz, Bernd Kersten, Benjamin Ulrich, Achim Elfering

**Affiliations:** 1 Institute of Psychology, University of Bern, Fabrikstrasse 8, 3012 Bern, Switzerland; 2 National Centre of Competence in Research (NCCR) Affective Sciences, University of Geneva, CISA, Chemin des Mines 9, 1202 Geneva, Switzerland; 3 Institute of Psychology, University of Leiden, P.O. Box 9555, 2300 RB Leiden, The Netherlands

**Keywords:** Relational devaluation, Social stressors, SOS-model, Health, Well-being

## Abstract

**Background:**

Social relationships are crucial for well-being and health, and considerable research has established social stressors as a risk for well-being and health. However, researchers have used many different constructs, and it is unclear if these are actually different or reflect a single overarching construct. Distinct patterns of associations with health/well-being would indicate separate constructs, similar patterns would indicate a common core construct, and remaining differences could be attributed to situational characteristics such as frequency or intensity. The current meta-analysis therefore investigated to what extent different social stressors show distinct (versus similar) patterns of associations with well-being and health.

**Methods:**

We meta-analysed 557 studies and investigated correlations between social stressors and outcomes in terms of health and well-being (e.g. burnout), attitudes (e.g. job satisfaction), and behaviour (e.g. counterproductive work behaviour). Moderator analyses were performed to determine if there were differences in associations depending on the nature of the stressor, the outcome, or both. To be included, studies had to be published in peer-reviewed journals in English or German; participants had to be employed at least 50% of a full-time equivalent (FTE).

**Results:**

The overall relation between social stressors and health/well-being was of medium size (*r* = −.30, *p* < .001). Type of social stressor and outcome category acted as moderators, with moderating effects being larger for outcomes than for stressors. The strongest effects emerged for job satisfaction, burnout, commitment, and counterproductive work behaviour. Type of stressor yielded a significant moderation, but differences in effect sizes for different stressors were rather small overall. Rather small effects were obtained for physical violence and sexual mistreatment, which is likely due to a restricted range because of rare occurrence and/or underreporting of such intense stressors.

**Conclusions:**

We propose integrating diverse social stressor constructs under the term “relational devaluation” and considering situational factors such as intensity or frequency to account for the remaining variance. Practical implications underscore the importance for supervisors to recognize relational devaluation in its many different forms and to avoid or minimize it as far as possible in order to prevent negative health-related outcomes for employees.

**Supplementary Information:**

The online version contains supplementary material available at 10.1186/s12889-021-10894-7.

## Background

Work is largely a social endeavour [1], and consequently many stressful experiences at work are social in nature [2, 3]. As will be elaborated below, there are many different concepts that reflect social stressors (e.g. aggression, incivility, or abusive supervision). However, in line with stress-as-offense-to-self theory [4], it can be argued that these different concepts have an important characteristic in common: They represent a threat to the self in terms of two strongly associated aspects of self-esteem, that is, social self-esteem (one’s perception of how one is evaluated by significant others) and personal self-esteem (one’s self-evaluation). This threat arises from feeling devalued (Leary & Allen [5] refer to “relational devaluation”), which violates the basic human need to belong ([6]; see also the need for relatedness in self-determination theory [7]). Relational devaluation may induce stress not only if directed against an employee as a person. Rather, people’s social roles, including their occupational roles, often become part of their identity [1] and therefore, part of their selves [4, 8–10]. It follows that a threat to the self may be induced by savaging occupational roles; such threats can be regarded as “identity-relevant stressors” [11].

Relational devaluation frequently leads to attempts to enhance and protect one’s self-esteem [12–14]; unless these are successful, emotional distress in the form of “dysphoric reactions are likely, such as depression, anxiety, jealousy, and hurt feelings” ([6] , p. 4), but anger-related reactions, somatic complaints, and other manifestations of low health and well-being are also typical [4]. The association of social stressors with health and well-being is the focus of the current meta-analysis, with a special emphasis on the question of the extent to which social stressors represent a common core construct (relational devaluation) or different constructs.

Social stressors are an issue of common interest in society. The prevalence of social stressors at work is noticeable. For example, the Swiss stress study [15] showed that 22% of the sample reported to have been exposed to social stressors within the previous 12 months.

In the UK [16] the number of cases with work-related stress, depression and anxiety in 2018/19 was 602,000 in total, corresponding to a prevalence rate of 1800 per 100,000 employees. The number of days lost per employee was estimated at 21.2 per year, corresponding to a loss of 12.8 million working days. Social stressors (violence, threats and bullying) accounted for 13% of the work-related stressors reported.

The European working conditions survey [17] showed that within 1 month prior to the survey 12% of employees experienced verbal abuse, 2% unwanted sexual attention, 6% humiliating behaviour and 4% threats. The percentage of employees reporting at least one adverse social behaviour differed between approximately 3 and 27% depending on the country. Those stressors were named as main reasons for staff turnover and absenteeism. Thus, empirical data indicate that social stressors represent a problem that is worth being taken seriously.

Although social stressors in terms of relational devaluation are rather common [18, 19], for a long time social stressors at work have received relatively little attention compared to stressors included in prominent models of stress research, such as workload. The first self-report scales on social stressors were introduced in German by Frese and Zapf [20], and in English as late as 1998 by Spector and Jex [21]; interpersonal conflict scale]. More recently, however, various research traditions have developed that focus on constructs akin to relational devaluation. Three approaches can be distinguished. One approach focuses on specific types of social stressors such as bullying [22] or abusive supervision [23, 24]; for an overview see [25]. A second group focuses on specific outcomes of social stressors such as aggression [26]. A third approach focuses on the argument that all kinds of social stressors may be subsumed under one term. For example, Bowling and Beehr [27] integrated different mistreatment variables under the term *workplace harassment* (abusive supervision, bullying, emotional abuse, generalised workplace abuse, incivility, interpersonal conflict, mobbing, social undermining, victimisation, workplace aggression), emanating from the assumption that all mistreatment variables refer to the same overall construct. Similarly, Hershcovis [28] conducted a meta-analysis in which she included different stressors in the category of *workplace aggression*, taking into account different moderator variables such as intent, intensity, or frequency. Both [27, 28] found effects similar in size, but there is also evidence for differences. For example, when looking at the source of aggression, Hershcovis and Barling [26] found strongest effects for aggression by supervisors, as compared to colleagues, on employee outcomes such as job satisfaction, affective commitment, intent to turnover, or psychological distress. Overall, it is not clear to what extent different stressors have different or similar effect sizes. Because most of the studies have focused on one specific stressor and different outcomes, or on different stressor constructs and one outcome, it is difficult to see the big picture. An important step towards integration is the paper by Hershcovis [28], who meta-analysed associations of five stressor constructs (incivility, supervisor aggression, bullying, interpersonal conflict, and social undermining) with various outcomes. Her results point more towards similarities than towards differences. However, a common core and specific differences may well exist simultaneously, as demonstrated by Baillien, Escartin, Gross, and Zapf [29], who showed that bullying/mobbing shares many features with other conflicts yet has very specific characteristics as well.

In the current meta-analysis, we aim to show commonalities and differences in the associations between various social stressors and a) well-being (emotional, physical, mental, general, burnout), b) behaviour (turnover intention, absenteeism, organisational citizenship behaviour, performance, counterproductive work behaviour), and c) attitudes (commitment, satisfaction). Our aim is to test whether results by Hershcovis [28] and Bowling and Beehr [27], which did not yield much support for specific patterns, can be supported using a comprehensive data set, or whether relations between stressors and outcomes are different, so that it is not justified to assume that all social stressors belong to an overall construct. This issue is important because it has implications for future research. If our results point to a predominance of commonalities among social stressors, research should more strongly focus on differentiating situational characteristics [29] when measuring social stress at work. If our results point to differences in stressor–outcome relations for different social stressors, the emphasis should be on identifying the most influential stressors and the most prominent outcomes for these different stressors. Thus, our results can lead to implications for work redesign and for interventions regarding social communication. At this point, we do not know of any meta-analysis comparing such a high number of social stressors and outcomes. Our intent is to broaden the knowledge about the relations between social stressors at work and various outcomes by expanding the number of potential associations.

### Social stressors

Sonnentag and Frese ([3], p. 562) defined social stressors in terms of “poor social interactions with direct supervisors, coworkers, and others”. Such social stressors convey devaluating social messages in a direct way. In addition, social stressors may also emanate from conditions at work. Thus, people can send “indirect social messages” through causing stress for others by being negligent (illegitimate stressors [30];), or by assigning tasks that people think should not have to be done or should be done by someone else (illegitimate tasks [31]).

In the literature, many different concepts of social stressors can be found; they include some key distinguishing features, but they also have considerable definitional, conceptual, and measurement overlap [28, 32, 33]. The most frequently used concepts of social stressors can be found in the supplementary file 1. Those behaviours can occur in varying intensity, duration or frequency, directedness, and intention [25, 28, 29, 34], which we refer to as situational characteristics. Besides these distinguishing situational characteristics, we contend that all social stressors have an important main feature in common: they are perceived as relational devaluation [5].

### Consequences of social stressors

The experience of relational devaluation through social stressors at work has been linked to various detrimental outcomes. In their meta-analysis, Hershcovis and Barling [26] categorised outcomes into three broad groups: health-related outcomes, behaviour, and attitudes. As the health-related outcomes typically refer to psychological and physical symptoms rather than to diagnosed illness, we will refer to these outcomes in terms of well-being in this article.

The following evidence is derived from several meta-analyses and reviews.

#### Well-being

Well-being is a multifaceted construct that can be addressed from physical, emotional, psychological, and mental perspectives [35]. Physical well-being is impaired by the experience of social stress at work [22, 26, 36]. Social stressors also influence emotional well-being in terms of high-arousal negative emotions (e.g. anxiety) [22, 27] and low-arousal negative emotions (e.g. depression) [22, 26, 37]. The impact of social stress on psychological and general well-being has been shown in terms of burnout [26, 27, 38] and general well-being [24, 28]. Further, the influence on mental health problems has been well established [22, 39, 40].

#### *Behavioural outcomes* (including behavioural intentions)

Bowling and Beehr’s [27] meta-analysis revealed that experiencing social stressors (summarised as harassment) increased turnover intentions. Similarly, increased absenteeism has been found [22]. Furthermore, task performance [23, 24, 26, 27] as well as organisational citizenship behaviour (OCB) [23] decreased by experiencing relational devaluation. On the other hand, counterproductive work behaviour (CWB), which describes a behaviour that negatively influences the productivity of an organization and/or its employees (e.g. withdrawing effort, leaving early, but also sabotage [41]) increased when experiencing destructive leadership [23, 24] or workplace harassment [27].

#### Attitudinal outcomes

Social stressors can affect attitudes such as commitment and satisfaction, as shown for workplace harassment [27], workplace bullying [22], and destructive leadership [24].

Table 1 displays recent findings from meta-analytic investigations.

**Table 1:** Integration of meta-analytic findings on social stress at work

Category	Facet	Abusive supervision (Mackey, Frieder, Brees, & Martinko, 2017)	Destructive leadership (Schyns & Schilling, 2013)	Destructive leadership (Montano, Reeske, Franke, & Hüffmeier, 2017)	Supervisor aggression (Hershcovis & Barling, 2010)	Supervisor-initiated aggression including abusive supervision (Hershcovis, 2011)	Co-worker aggression (Hershcovis & Barling, 2010)	Outsider aggression (Hershcovis & Barling, 2010)		
Well-Being	Emotion: high arousal – negative									
Well-Being	Emotion: low arousal – negative	.21*			.24*		.18*	.38*		
Well-Being	physical				.15*	.15*	.20*	.17*		
Well-Being	mental				.25*	.30*-.31*	.19*	.19*		
Well-Being	Burnout	.32*		.31*	.30*		.25*	.31*		
Well-Being	general		.01–.37*	.27*						
Behaviour	Turnover intention		.22*-.34*		.26*	.30*	.20*	.15*		
Behaviour	Absenteeism									
Behaviour	Organisational Citizenship Behaviour (OCB)	.21*								
Behaviour	Performance	.17*	.18* − .22*		.15*		.07*			
Behaviour	Counterproductive Work Behaviour (CWB)	.31*-.48*	.29*-.40*		.29*-.34*		.25*-.38*	.18*-.24*		
Attitude	Commitment	.23*	.19*-.28*		.24*	.24*-.26*	.17*	.07*		
Attitude	Job satisfaction	.31*	.27*-.35*		.32*	.34*-.35*	.20*	.12*		
Attitude	Life satisfaction									
Category	Facet	Interpersonal conflict (Hershcovis, 2011)	Interpersonal conflict (Nixon, Mazzola, Bauer, Krueger, & Spector, 2011)	Incivility (Hershcovis, 2011)	Role ambiguity (Alcaron, 2011)	Role ambiguity (Nixon, Mazzola, Bauer, Krueger & Spector, 2011)	Role ambiguity (Schmidt, Roesler, Kusserow, & Rau, 2014)	Role conflict (Nixon, Mazzola, Bauer, Krueger, & Spector, 2011)	Role conflict (Schmidt, Roesler, Kusserow, & Rau, 2014)	Role conflict (Alcaron, 2011)
Well-Being	Emotion: high arousal – negative									
Well-Being	Emotion: low arousal – negative						.28*		.29*	
Well-Being	physical	.16*	.22*	.17*		.15*		.27*		
Well-Being	mental	.35*		.33*						
Well-Being	Burnout				.24*-.26*					.29*-.42*
Well-Being	general									
Behaviour	Turnover intention	.33*		.36*						
Behaviour	Absenteeism									
Behaviour	Organisational Citizenship Behaviour (OCB)									
Behaviour	Performance									
Behaviour	Counterproductive Work Behaviour (CWB)									
Attitude	Commitment	.21*		.31*						
Attitude	Job satisfaction	.29*		.40*						
Attitude	Life satisfaction									
Category	Facette	Workplace discrimination (Dhanani, Beus, & Joseph, 2018)	Discrimination (Jones, Peddie, Gilrane, King, & Gray, 2016)	Workplace bullying (Nielsen, Indregard, & Øverland, 2016)	Workplace bullying (Nielsen & Einarsen, 2012)	Bullying (Hershcovis, 2011)	Workplace harassment (Bowling & Beehr, 2006)	Work harassment (Sojo, Wood, & Genat, 2016)	Sexual harassment (Sojo, Wood, & Genat, 2016)	
Well-Being	Emotion: high arousal – negative				.27*		.25*			
Well-Being	Emotion: low arousal – negative				.34*		.28*			
Well-Being	physical	.19*^a^	.13*		.10–.28*	.32*	.25*		.17*	
Well-Being	mental	.29*^a^	.25*		.20*-.34*	.40*		.37*	.27*	
Well-Being	Burnout				.27*		.33*			
Well-Being	general				.31*-.37*				.23*	
Behaviour	Turnover intention				.28*	.35*	.29*			
Behaviour	Absenteeism			.13*	.11*-.12*		.06			
Behaviour	Organisational Citizenship Behaviour (OCB)						.02			
Behaviour	Performance				.12		.06			
Behaviour	Counterproductive Work Behaviour (CWB)						.30*			
Attitude	Commitment				.19*		.30*			
Attitude	Job satisfaction				.22*	.39*	.32*			
Attitude	Life satisfaction						.18*			

Note. ^a^ = rho, correlation corrected for artefacts; all effects are corrected for their direction stressor-impairment (i.e., positive correlations imply that stressors are associated with lower well-being/health); empty cells indicate that no coefficients were reported for the specific combination of stressors and well-being/health; * *p* < .05

As the table shows, most of the meta-analytic studies have investigated the effect of a specific social stressor on different outcomes. While effects within studies do differ, the results reflect a certain pattern.

Some outcome variables show significant associations across stressors rather consistently. These are negative emotions (both high and low arousal), mental well-being, burnout, general well-being, turnover intention, and attitudinal outcomes (commitment, job satisfaction). An exception is the correlation of .01 for destructive leadership and general well-being, but that is based on k = 1 with an N of 67. These outcomes reflect mainly psychological well-being and job/organization-related attitudes/intentions.

Another group of outcomes shows associations that differ more strongly depending on the specific stressors investigated, although, with one exception, all correlations are significant. These are physical well-being, OCB, performance, and counterproductive work behaviour, which seem to differ depending on the specific stressor. Note that behavioural outcomes dominate this category.

Finally, for absenteeism and life satisfaction, only few effects have been reported, and these were rather small.

From these meta-analyses, we can conclude that experiencing social stressors is associated with well-being, behaviour, and attitudes in the expected direction. There are commonalities and differences, the former relating mostly to psychological reactions, the latter to physical reactions and behaviour. The reason for this pattern might be that affective reactions represent rather direct effects, whereas behaviour depends on many additional mechanisms, such as forming intentions and encountering circumstances that allow for behaviours such as counterproductive work behaviour. It is debatable, however, whether the differences in these data arise from real differences between the social-stressor variables. To investigate differences and commonalities in associations between specific stressors and outcomes, we therefore will discriminate among social stressor-variables as well as outcomes in our meta-analysis. We will use a comprehensive data set with sufficient power to detect possible difference.

## Methods

### Literature search

We conducted a systematic literature search using PsycINFO, PSYNDEXplus Lit. & AV, PSYNDEXplus Tests, and MEDLINE, and additionally Web of Science and Ebscohost (including Business Source Premier, CINAHL, MEDLINE, and SocINDEX). In addition, articles and reference lists of earlier meta-analyses and systematic reviews were screened for pertinent articles. Further, we contacted researchers in the field to contribute additional studies. Published articles that we could not access were requested from the authors. The research project was carried out in three waves in 2007, 2012, and 2015. During these three steps we found a total of 557 studies published before 2015 that met our inclusion criteria (the whole selection process is imaged in Supplement 2 according to PRISMA guidelines [42]). After an identification search including the databases listed above and additional references found by reference list screening or added by researchers in the field, duplicates were removed. In a second step the records found were screened for their title and abstract. Following, full-texts were screened for their eligibility and, if suitable, included in our analysis.

### Inclusion and exclusion criteria

In accordance with the PICOS framework (see Supplement 4 [43]), the studies had to report a correlation between social stressors at work and well-being/health, attitudes, or behavioural measures to be included. For studies lacking information, we requested additional information. In case of no response by the authors, the studies were excluded.

To ensure a substantial exposure to social interaction at the workplace, the sample had to consist of employees working 50% or more of a full-time equivalent (FTE). If samples were used twice or more for publishing, but focused on different variables, those articles using the same sample were aggregated into one sample. If the same variables were used in different articles based on the same sample, we chose one article randomly for inclusion. Further, the studies had to be published in peer-reviewed journals in German or English and contain quantitative data. We chose only published studies to ensure satisfactory scientific quality; we did not include dissertations. Student samples could be included if working hours were at least 50% of a FTE. We did not include military workers, children, non-working adults, pensioners, and clinical samples. In Supplement 5 we provide a full list of all references included in the analysis.

### Coding

For each of the three waves of data collection, two independent raters coded the studies. The codes refer to different categories. On the one hand, they entailed correlation coefficients. Furthermore, the independent variables were coded in terms of specific social stressors at the workplace (e.g. mobbing, bullying, abusive supervision, incivility); the dependent variables included well-being (e.g. emotions, physical well-being, mental well-being), behavioural (e.g. CWB, turnover intention, performance) or attitudinal outcomes (commitment, satisfaction) in accordance with the definition in the PICOS framework (see Supplement 4). Further codes refer to sample, study quality, measurement, and possible moderators. Overall, interrater reliability reached ĸ = .74, which is in the upper range of what can be considered a moderate level of consensus [44]. Note that the coding included qualitative data (e.g. description of methods), for which very high reliabilities are more difficult to obtain. For study quality a score was generated including sample size, response rate, and reliability of both independent and dependent variable, which was used as a control variable in the analysis; no significant difference between the different studies in terms of study quality emerged.

### Meta-analytic calculations

Before starting an analysis, the results of studies need to be converted to a common standardised effect size measure, which makes comparison and synthesis of the data possible [45]. We coded correlational data or transformed other data to correlations. Next, we combined variables into broader categories. For the independent variables, social stressors were grouped by characteristics such as type of behaviour, source of stress, and measurement instrument. Following Hershcovis and Barling [26], the dependent variables were grouped as reflecting well-being, behaviour, or attitudes. Because multiple effects can occur per study (e.g. a correlation of bullying and incivility with performance within the same sample), we need to take into account that those are more alike than other effects. Because multivariate models are often inapplicable due to the lack of information about correlations between effect sizes [46–48] we used overall effects for each study by calculating the average of all included effect sizes per study (which means we averaged the correlation coefficients available in each study). When there were similar measures of the same construct or group of independent variables in one study (e.g. two different measurement instruments for burnout), it can be argued that the population effect sizes are the same. Therefore, it makes sense to use the average of the observed effect sizes within a study as the observed effect from this study further on in the meta-analysis [49] (see Tables 2 and 3 for dependent and Table 4 for independent variables). A weighted mean can be used when the sample size varies over the different findings within a study, so that effect sizes based on larger samples get more weight. Just like in a standard meta-analysis, the inverse of the variance can be chosen as the weight, minimising the sampling variance of the average effect.

For our calculations, we used the program Comprehensive Meta-Analysis (CMA) [50]. The overall effect size for all studies was calculated as the mean of the averaged correlations across samples, weighting each observed correlation by the study’s sample size. Q-statistics were used to represent the heterogeneity of the studies. When the Q-value is significant, the null hypothesis of homogeneity is rejected. An I^2^ statistic was computed as an indicator of heterogeneity in percentages, with increasing values indicating increasing heterogeneity. Because we expected heterogeneity between studies to occur, we used the random effects model for calculating effect sizes. This approach is recommended when there is reason to assume that the measured effect sizes in studies vary, and when it is improbable that studies are identical [51].

### Main effects

First, we calculated an overall mean effect representing the general association between social stressors and the well-being/health indicators. We then calculated main effects for each outcome variable (high-arousal negative emotions, low-arousal negative emotions, physical well-being, mental well-being, burnout, general well-being, turnover intentions, absenteeism, OCB, performance, CWB, commitment, life satisfaction, and job satisfaction) and for each group of outcome variables (well-being, behaviour, and attitudes), so the patterns could be compared. Effects similar in size would point to a common core construct. Effects with coherent directions but different sizes might imply a common fundamental construct with different intensity, frequency, etc. To see if there are specific patterns, we calculated all cross-correlations between predictors (social stressors) and outcome variables. Clear patterns, that is, specific predictors only correlating with certain outcomes, would point to different constructs rather than a common fundamental construct.

### Moderator analyses

To see if there are differences in the associations between various stressors and outcomes, we first wanted to see if the type of independent or dependent variable moderated the main effect. Therefore, we created categorical moderator variables. For example, each dependent variable received a number that was used as the categorical moderator (well-being: high-arousal negative emotions = 1, low-arousal negative emotions = 2, physical well-being = 3, mental well-being = 4, burnout = 5, general well-being = 6, behaviour: turnover intention = 7, absenteeism = 8, OCB = 9, performance = 10, CWB = 11; attitudes: commitment = 12, life satisfaction = 13, job satisfaction = 14). The same procedure was applied to the independent variables. When doing a subgroup analysis based on the type of variable (as a categorical moderator), one can choose between different options (see CMA manuals) [52], each having advantages as well as disadvantages. Using CMA [50], there are three different approaches: First, one can group by moderator variable and compare the different groups by using their *averaged* effect. This method is convenient to use, but because different moderator values can occur within one sample (e.g. turnover intention as well as performance), averaging them will not yield a meaningful outcome referring to the difference between variables. Therefore, several samples will belong to a category that is not clearly associated with a moderator value and is hence not interpretable. The second approach is to use all the outcomes assuming *independence*. This would include all information in the data set, but dependencies between effect sizes within the same sample would not be taken into account, which can lead to imprecision as well as wrong estimates of variance (see CMA manuals). A third possibility is to include only *one randomly selected* effect per sample. In case there are several categories of stressor variables present within one sample, one category will be selected randomly. Using this approach, one would lose some information but could clearly interpret every moderator group. Weighing the pros and cons of the different approaches, we applied the approach of randomly selected effects.

To decide whether to conduct moderator analysis, the procedure proposed by Hedges and Olkin [46] was applied. A significant Q_B_ points to the presence of a moderator, suggesting a difference among the mean effect sizes across the groups tested. Following a benchmark set by Nielsen and Einarsen [22], we conducted moderator and subgroup analysis when significant Q_B_’s were present at least within three different samples.

Because overall effect sizes can be overestimated due to publication bias in favour of significant findings, we calculated the fail-safe N as an indicator of robustness, which reflects the number of studies reporting null results that would be required to reduce the overall effect to non-significance [49].

### Dealing with multiple comparisons

Referring to multiple comparisons within such a broad data set, one would typically use a method of type 1 error control to prevent an inflation of the α-error. Following Keselman, Miller, and Holland [53], such control procedures seem reasonable when the number of tests is about 15 and above. Traditional approaches like the Bonferroni procedure [54–56] belong to the category of family-wise error rate-controls (FWER) [53], which get very restrictive and insufficient in detecting non-null hypotheses with an increasing number of hypotheses to be tested [57]. Therefore, new methods have been introduced that are more powerful in detecting false null hypotheses by allowing the researcher to reject a defined number of true null hypotheses (k-FWER) [57]. Keselman and colleagues [53] reported a comparison of those newer methods based on two data sets derived from the published literature. Results show that fewer null hypotheses were rejected with those k-FWER methods than without any correction, and more than the traditional FWER, but the number of detected effects differed among the different approaches depending on the procedure used. The Cochrane Scientific Committee [58] is also aware of the problem of the “increased probability of rejection of the null hypothesis on repeated meta-analysis”. The committee discussed possible methods for correction based on the work of Mark Simmonds [59] showing that a control on type 1 error seems overly conservative and is therefore not recommended.

Because we are analysing a very comprehensive data set with multiple comparisons within our cross-correlation calculations, we prefer to be on the conservative side, potentially missing non-null hypotheses rather than accepting false effects. Therefore, we did not apply k-FWER methods [57]. On the other hand, a Bonferroni correction would be, indeed, overly conservative. We therefore decided for a middle ground by adapting the level of significance to α = .01 to avoid type 1 errors.

## Results

### Description of the studies included

We integrated 557 studies, including 640 different samples, which yields an overall *N* of 387,407 participants. The largest number of samples (289) was from the United States of America and Canada (45.2%). A total of 151 studies (23.6%) were from Central Europe, 54 studies (8.4%) were from Asian countries, and 103 studies (16.1%) were carried out in other countries (e.g. Slovenia, Romania, Pakistan). For 43 studies (6.7%), no information on the country of origin was provided. The publishing date of the studies displays a clear trend. While 5.6% of the included studies were published prior to 1995 and 28.4% before 2005, 65.2% of the studies were published from 2006 on. On average, the samples consisted of 55.5% female participants. The mean age was 36 years (*SD* = 8.90). The average response rate was 58.5%, which accords with the average response rate for organisational surveys [60].

### Weighted mean correlations

The overall weighted mean correlation between social stressors overall and the combined outcomes was *r* = −.30 (95% CI = −.31, −.28, *p* < .001), representing a moderate effect. Because indicators for heterogeneity were high (*Q* = 10,924.98, *p* < .001; *I*^*2*^ = 94.15), we tested whether the type of outcome or group of stressors showed a moderation effect. Both the type of outcome (*Q* = 85.96, *p* < .001) and group of stressors (*Q* = 48.44, *p* < .001) explained a significant amount of variance and therefore acted as moderators.

The weighted mean correlations between social stressors overall and the individual outcomes are displayed in Table 2.

**Table 2:** Meta-analysis of work-related outcomes of experienced social stress at work (random effects model)

Outcome	*K*_S_	Mean *r*	95% CI	*Q*	*I*^2^	NMS
High arousal – negative	38	.29***	.26; .32	94.72***	6.94	11,342
Low arousal – negative	56	.30***	.27; .33	378.50***	85.47	30,190
Physical	105	-.22***	−.25; −.19	1685.71***	93.83	50,820
Mental	88	−.27***	−.30; −.23	122.41***	92.87	62,617
Burnout	76	.34***	.31; .37	495.46***	84.86	61,164
General Well-Being	44	−.25***	−.29; −.21	405.84***	89.41	9547
Turnover Intention	208	.27***	.25; .29	2465.77***	91.61	370,753
Absenteeism	22	.12***	.09; .15	77.94***	73.06	1535
OCB	103	−.22***	−.25; −.19	797.16***	87.21	34,337
Performance	88	−.22***	−.25; −.19	844.31***	89.70	31,053
CWB	88	.30***	.26; .34	1218.88***	92.86	57,452
Commitment	150	−.34***	−.37; −.31	2394.46***	93.78	238,850
Life satisfaction	21	−.14***	−.17; −.11	64.19***	68.84	1626
Job satisfaction	272	−.36***	−.38; −.34	5305.19***	94.89	961,890

Note. *** *p* < .001

*K*_S_ = number of samples; Mean *r* = weighted mean correlation coefficient; 95% *CI* lower and upper limits of 95% confidence interval for mean *r*; *Q* = indicator of heterogeneity; *I*^*2*^ = indicator of heterogeneity in percentages, *NMS* number of missing studies that would bring the *p* value to > alpha, based on the fail-safe N method

Social stressors were significantly related to all outcomes referring to well-being (high-arousal negative, low-arousal negative, physical, mental, burnout, general), behavioural characteristics (turnover intention, absenteeism, OCB, performance, counterproductive work behaviour), and attitudinal outcomes (commitment, life satisfaction, job satisfaction). Following Cohen’s conventions [61], all associations except absenteeism and life satisfaction were about moderate in size. Both absenteeism and life satisfaction were represented by comparably few samples, which makes the results more likely to be influenced by possible outliers. Especially emotional exhaustion (burnout), commitment, and job satisfaction showed comparably high relations with experienced social stress at work.

To see whether associations with our three categories were different from each other, we additionally compared well-being, behavioural, and attitudinal outcomes (Table 3). Because life satisfaction was represented only by three samples, we treated it separately and, due to the non-significant relation, excluded it from further analysis. The effects on well-being and behavioural outcomes were small to moderate in size, whereas attitudinal outcomes reached a moderate effect size. We found a significant moderation effect showing that the three outcomes were significantly different from each other.

**Table 3:** Meta-analysis of work-related outcome categories of experienced social stress at work (random effects model)

Outcome	*K*_S_	Mean *r*	95% CI	*Q*_*B*_	*df*	*p*	*Q*_*B*_	*df*	*p*
Well-being	183	−.29***	−.31; −.27	47.75	3	.00	43.53	2	.00
Behaviour	247	−.26***	−.27; −.24						
Attitudes	207	−.35***	−.37; −.33						
Life satisfaction	3	−.12	−.30; .06						

Note. *** *p* < .001; all reported effect sizes are corrected for their direction

*K*_S_ = number of samples; *M*ean *r* = weighted mean correlation coefficient; 95% *CI* lower and upper limits of 95% confidence interval for mean *r*; *Q*_*B*_ indicator of moderation effect; *df* degrees of freedom

Because we also found a significant moderation effect of type of social stressor on the overall mean effect, we calculated the main effects for every type of stressor (Table 4). We did not consider effect sizes that were based on a single study only (identity threat and stereotype threat). Effect sizes were moderate for mobbing/bullying, incivility, identity threat, stereotype threat, undermining, illegitimate tasks, and justice; all other effects were small to moderate in size. Among the social stressors represented by a sufficient number of studies (at least three samples included), lack of justice seemed to show the highest effects, followed by incivility and mobbing/bullying.

**Table 4:** Main effects for different social stressor types (random effects model)

Outcome	*K*_S_	Mean *r*	95% CI	*Q*	*I*^2^	NMS
Role stress	63	−.25***	−.29; −.21	746.74***	91.70	29,837
Interpers. conflicts	135	−.29***	−.31; −.26	1782.72***	92.48	143,344
Conflict with supervisor	11	−.25***	−.33; −.16	42.85***	76.66	389
Conflict with clients	10	−.27***	−.38; −.15	121.37***	92.58	618
Perceived victimisation	5	−.24***	−.28; −.20	5.16	22.40	265
Supervisor mistreatment	42	−.26***	−.30; −.22	257.98***	84.11	8272
Mobbing/Bullying	62	−.32***	−.35; −.29	1289.21***	95.27	70,354
Social exclusion	13	−.29***	−.38; −.19	128.92***	9.69	1114
Sexual mistreatment	42	−.19***	−.23; −.15	277.98***	85.25	5509
Harassment	22	−.24***	−.30: −.17	298.03***	92.95	2988
Incivility	40	−.32***	−.36; −.29	226.84***	82.81	15,305
Identity threat	1	−.39***	−.50; −.27	na	na	na
Physical violence	19	−.17***	−.22; −.11	86.86***	79.28	833
Verbal/emotional violence	21	−.25***	−.30; −.20	77.57***	74.22	2040
Mistreatment	41	−.24***	−.28; −.19	538.18***	92.57	9575
Stereotype threat	1	−.46***	−.55; −.36	na	na	na
Hostility	7	−.25***	−.29;-.20	92.10***	93.49	2654
Undermining	5	−.18**	−.30; −.05	51.54***	92.24	69
Illegitimate tasks	2	−.29***	−.37; −.19	.96	na	na
Lack of justice	239	−.33***	−.36; −.31	5531.28***	95.70	466,216

Note. ** *p* < .01; *** *p* < .001; all reported effect sizes are corrected for their direction

*K*_S_ number of samples; *M*ean *r* weighted mean correlation coefficient; 95% CI = lower and upper limits of 95% confidence interval for mean *r*; *Q* indicator of heterogeneity; *I*^*2*^ indicator of heterogeneity in percentages, *NMS* number of missing studies that would bring the *p* value to > alpha, based on the fail-safe *N* method; all effects are corrected for effect direction, representing a stressor-health relationship; *na* not available

Because we found significant moderation effects for type of social stressor as well as for outcome category, we calculated cross-correlations representing every possible combination of stressor and outcome that was based on at least three samples. These cross-correlations are summarised in Table 5.

**Table 5:** Cross-calculations for stressor-outcome relationships

	Well-Being						Behaviour					Attitudes	
	Emotions		Physical	Mental	Burnout	General	Turnover	Absenteeism	OCB	Performance	CWB	Commitment	Job satisfaction
	High arousal negative	Low arousal negative											
Role stress			−.23**				.32***		−.21*	−.25***		−.25***	−.40***
Interp. conflicts	.32***	.31***	−.22***	−.25***	.33***	−.31***	.28***	.11**	−.14*	−.13*	.42***	−.23***	−.30***
Supervisor mistreatment				−.61***	.27***		.27***		−.23***	−.16***	.29***	−.24***	−.39***
Mobbing and bullying	.30***	.35***	−.28***	−.31***	.41***	−.21***	.28***	.14***		−.17		−.35***	−.32***
Social exclusion							.24***			−.33***	.48***		−.26***
Sexual mistreatment	.30***	.22***	−.14***	−.20***			.17***				.23***	−.10	−.23***
Harassment			−.17*	−.17**			.19***				.43***	−.24***	−.28***
Incivility		.35***	−.32***	−.36***	.36***		.30***				.22***	−.27***	−.34***
Physical violence			−.16***	−.10	.26***								−.12
Verbal & emotional violence					.28***		.21***					−.16	−.35***
Mistreatment, aggression, and violence		.23***	−.20***	−.32***	.32***	−.10	.20***						−.27***
Hostility							.18***						−.18**
Lack of justice	.25***	.26***	−.14	−.28***	.34***		.30***	.11***	−.24***	−.25***	.19***	−.42***	−.45***
Moderator analysis (*p*)	.55	.12	.04	.00	.33	.01	.01	.67	.49	.15	.00	.00	.00

Note. * *p* < .05; ** *p* < .01; *** *p* < .001; all cross-correlations are represented by at least 3 samples; *OCB* organisational citizenship behaviour; *CWB* counterproductive work behaviour

Coefficients differed widely, ranging from .10 to .61, representing small to almost large correlation coefficients. Because we already showed that there was a significant difference between the distinct outcomes (Table 3), we conducted another moderation analysis of the type of social stressor within all categories. For high-arousal negative emotions, low-arousal negative emotions, burnout, absenteeism, OCB, and performance, there was no difference in the size of correlations with respect to the type of social stressor. In contrast, within physical, mental, and general well-being, as well as turnover, CWB, commitment, and job satisfaction, the type of social stressor did moderate the effect size. For example, within physical well-being, incivility showed the strongest effects, followed by mobbing/bullying, whereas lack of justice did not reach statistical significance. On the other hand, within the two attitudinal outcomes commitment and job satisfaction, lack of justice reached the strongest effects. Thus, depending on the outcome, the type of social stressor matters.

### Publication bias

Because in meta-analyses effect sizes can be overestimated concerning publication bias in favour of significant findings, we calculated the classic fail-safe N, which shows the number of studies reporting null findings that would be needed to bring the significant mean effect to non-significance [49]. For all mean effects, the fail-safe N estimates showed that the calculated effect sizes are likely to be steady (see Tables 2 and 4).

## Discussion

Within the present meta-analysis, 557 studies comprising 640 different samples were analysed with respect to the association of social stressors at work with well-being and health-related outcomes. We found an overall relation of *r* = −.30, representing a moderate correlation. To deal with the heterogeneity of the results, we grouped the outcome variables into the three categories well-being, behaviour, and attitudes. All three categories were affected by social stressors at work, but their effects differed significantly. Furthermore, social stressors differed significantly when analysed as a moderator within the overall mean effect. Due to the comprehensive sample including various social stressor constructs, a notable amount of heterogeneity arose. Within this variance, the outcome categories well-being, behaviour, and attitudes were able to explain a significant amount of variance. Nevertheless, when comparing the size of the correlation effects of the three groups, they are comparable with regard to Cohen’s conventions [61].

The overall mean correlation of social stressors and health- and well-being-related outcomes supports the existing evidence [22, 27, 28]. The effects differed by both type of social stressor and outcome category. Differences concerning outcome variables (well-being, behaviour, attitudes) show a clear pattern, with attitudinal outcomes such as commitment and job satisfaction showing the highest effects. This also supports earlier research [28]. In addition to commitment and job satisfaction, emotional exhaustion (burnout) and counterproductive work behaviour also yielded moderate effect sizes. Surprisingly, there was a comparatively low effect for absenteeism, which might be due to fewer samples contributing to this effect. However, Nielsen and Einarsen [22] and Nielsen, Indregard, and Øverland [62] found effects of bullying on absenteeism comparable to the one we found. A problem with studies on absenteeism is that it is often not clear whether only voluntary absenteeism is included, or also absenteeism due to sickness [63]. Physical outcomes show comparatively low associations with stressors in our study, and also in research on stress at work on the whole [64]. In general, all outcome categories reached significance and are therefore associated with social stressors at work. The fact that attitudinal outcomes are concerned most might show the influence on short-term outcomes. It is conceivable that after an attitude has developed it might lead to behavioural intentions or acts, for instance in terms of revenge [65, 66], implying that an individual is more likely to engage in negative behaviours as compensation tit for tat [67]); however, engaging in such behaviours requires additional steps and decisions, implying lower associations as compared to attitudes.

When looking at the mean effects of the different social stressor constructs, we can also see a variety of effects indicated by a significant amount of heterogeneity; however, while statistically significant, the differences between effect sizes are not very large. Lack of justice shows the strongest effects, followed by incivility and mobbing and bullying. Unfortunately, stereotype threat, identity threat, illegitimate tasks, undermining, perceived victimisation, and hostility were represented by comparatively few samples. It is therefore difficult to draw conclusions regarding these variables. Overall, the variance in effect sizes between different social stressor concepts is smaller as compared to those of the outcomes. Put differently, the effects of different social stressors are more similar than the general effect of social stress on different outcomes.

To be able to draw more specific conclusions regarding distinct relations, we calculated cross-correlations, which showed certain patterns. First, there is one group of outcome variables for which the effects of distinct social stressors are not different. This group contains negative emotions, both high arousal and low arousal, burnout, absenteeism, OCB, and performance. Second, we have a group of outcomes for which the type of social stressor matters with respect to the effect size. This category includes physical, mental, and general well-being, turnover, CWB, commitment, and job satisfaction. This indicates that there are indeed distinct patterns for social stressors and strain relations, and that it might be important to distinguish the kind of devaluation experienced with regard to the outcome of interest. It seems striking that lack of justice, supervisor mistreatment, and mobbing/bullying are especially important for attitudes. All three constructs are characterised by an imbalance of power, which underscores the importance of this aspect. Furthermore, harassment, social exclusion, and interpersonal conflicts seem to be important especially when measuring counterproductive work behaviour. What also striked us is that incivility shows comparatively high effects on all outcomes. Even though it is defined as a low-intensity behaviour of ambiguous intent [67], it is associated with health and well-being to a much higher extent than more obviously threatening stressors (e.g. mistreatment, aggression, and violence), which might be due to its higher occurrence compared to high-intensity behaviours.

### Range restriction regarding specific social stressors

When looking only at the strength of the social stressor concepts with sufficient sample sizes across different outcomes (Table 4), we see that most of them are similar in size, suggesting the presence of an overall construct. The largest effect sizes concern lack of justice (*r* = −.33), incivility (*r* = −.32), and mobbing/bullying (*r* = .32), representing moderate effect sizes. Most of the other concepts yield effect sizes ranging between .24 and .29, representing small to moderate effects. Smaller effect sizes are found only for sexual mistreatment (*r* = −.19), physical violence (*r* = −.17), and undermining (*r* = .18), the latter being represented by a fairly small number of studies. It is striking that both sexual mistreatment and physical violence not only express relational devaluation but also threaten and violate a person’s physical integrity. Both concepts have a very high intensity, and they likely occur less often compared to lower-intensity behaviour, such as incivility. Consequently, they might show restricted variance when compared to stressors occurring more often. This would explain their lower effect size despite their high intensity. Additionally, sexual mistreatment might especially suffer from underreporting due to victims not reporting each incident that might have happened. This would further support our assumption of restricted variance in those concepts. Rare reporting, be it due to rare occurrence and/or to underreporting, induces skewness in the data and reduces the maximum correlation possible. If that is taken into account, the values obtained for variables that are reported infrequently may well be erroneous in suggesting a low association. More specifically, in such cases low-correlation coefficients can be associated with high relative risks associated with the occurrence of rare social stressors (cf. the correlation of *r* = .10 between smoking and lung cancer, which implies a relative risk of 11) ([68] , pp. 53–54).

The similarity in effect sizes across different social stressors and different outcomes suggests that there is, indeed, a common core to social stressors; we feel that the term “relational devaluation” describes this core very well. Differences would then mainly be due not to a different nature of the different stressors but to additional characteristics mentioned in the literature. The most prominent examples of such additional characteristics are duration/repetitiveness, intensity, and power differential [25, 28, 29, 34]. Such distinguishing features might be responsible for the heterogeneity found, as well as for the restriction of variance in high-intensity/low-frequency constructs, such as physical violence. As an example, offensive behaviours such as ridiculing, excluding, and insulting, which characterise mobbing, may well occur as social stressors yet not be characterised as mobbing if they are infrequent, experienced over short periods of time (e.g. at times in which a superior is stressed), and addressing not a specific target but whoever happens to be around [69, 70]. Thus, it is the specific additional characteristics of social stressors in a given context, rather than their intrinsic qualities, that are likely responsible for differences in effect sizes. The validity of this conclusion can be determined, however, only if these additional characteristics are assessed simultaneously; only then is it possible to determine if such characteristics play a decisive role in predicting outcomes—and if they do, in which constellations. We therefore suggest that future studies assess as many of these characteristics as possible.

Following Berry and colleagues [71], we were able to show that the existing empirical data show patterns, but the differences found were predominantly on the outcome level. This means we mainly found differences in effect sizes depending on the measured outcomes. Our data indicate that overall social stressors representing relational devaluation should be taken into account as a serious threat to well-being and health. For organisations, it should be interesting that lack of justice, supervisor mistreatment, and mobbing/bullying show especially high associations. Note that all three of these are typically characterised by a high power differential. All three of them can be targeted by organisational policies to prevent them or to raise awareness. Supervisor trainings can be tools to optimize leadership behaviour and to sensitise leaders for employee needs. Furthermore, the fact that attitudes might be affected most easily by social stress at work yields a good diagnostic tool for organisations. If they consistently ask about their employees’ satisfaction, they will have a good indicator for interpersonal as well as climate problems. Because we found that all investigated social stressor concepts show an impact, it might be appropriate for organisations to use more general measurement instruments to screen for relational devaluation. In case of specific problems within the organisation, more specified measurement instruments could be used, depending on the situational characteristics. Our results indicate that low-intensity but high-frequency behaviours (e.g. incivility) should not be underestimated. At the same time, the comparatively low associations with outcome variables of high-intensity but low-frequency behaviours, such as physical aggression or sexual offenses, should not be mistaken to imply a low impact, as low frequency and underreporting restrict variance, and thus the maximum correlation that can be obtained.

For future research, it is important to investigate long-term effects. It would be interesting to see if the effects change during long-term assessments, with stronger effects for well-being-related outcomes due to the consistency of social stressors over time (versus adaptation). Furthermore, it would be interesting to have a closer look at our finding of differences in terms of outcomes but much smaller differences between social stressor constructs. Why are the effects of social stressors different within some outcomes and not within others? Moreover, the consideration of moderating situational variables, such as power balance, source of stress (customer, colleague, and supervisor), duration, and intensity, seems to be promising. Further, gender and ethnicity might play a role as well. As the European working conditions survey [17] pointed out, depending on the adverse social behaviour investigated, there seem to be differences relating to gender. For example, more men are targeted by threatening behaviour. Following, it could be promising to explore the effects for men and woman separately, which is not often done in existing research. As has been mentioned in the introduction the numbers of adverse social behaviour differ a lot depending on the country, which might point to cultural differences. A completely new field in the area of social stressors is arising with research on human–machine interactions [72]. So-called “hybrid teams” consisting of at least one human and one machine agent can also be expected to be exposed to social stress, for instance when machine agents take over tasks typically carried out by a human agent. It seems promising to focus more strongly on this research and draw comparisons between human and machine agents.

### Limitations

The present meta-analysis has some limitations. First, we did not include studies published in books, dissertations, or “grey” literature, such as research reports. Second, we included a broad variety of different types of social stressors as well as well-being- and health-related outcomes to be able to compare them. Therefore, we had to face a highly heterogeneous data set. We tried to deal with this issue by grouping social stressors as well as outcomes in categories derived from the literature. Third, due to our strategy for cross-calculations, we randomly selected one effect per study to ensure interpretable results. Therefore, not all evidence has been integrated within the cross-calculations. However, due to the random selection there should be no bias. Fourth, the effects were mainly cross-sectional, preventing us from drawing causal conclusions and conclusions regarding long-term effects. Investigating long-term effects certainly seems warranted. As mentioned above, we did not include research on human–machine interactions but focused on human interactions exclusively. Further, effects could not be analysed separately for men and women, as such analyses are seldom reported in the literature, and using the percentage of males/females as a moderator would not be more than a very rough proxy.

On the other hand, our study has several strengths. First, we included a broad variety of different social stressors as well as well-being- and health-related outcomes to be able to compare them. By building classifications and testing for potential moderators, we tackled the existing heterogeneity. Due to our cross-correlations, we were able to show unique patterns that have not been shown before. Second, we included 557 studies representing 640 different samples, which is a comparatively high number. We do not know of any meta-analytic study in the field integrating such a great amount of data. This should result in high power to find results (although it also entails the danger of chance findings, which we dealt with by applying a strict criterion for statistical significance). Third, we applied meta-analytic calculations to quantify our findings.

## Conclusions

Despite the large heterogeneity we had to face, we were able to integrate a comparatively large number of 557 studies representing 640 samples within a categorical framework of dependent variables derived from the literature. We could support earlier research by showing an overall moderate effect of social stressors on well-being and health and by demonstrating that social stressors overall have an effect, with both the type of social stressors as well as outcomes accounting for a significant amount of variance but with more heterogeneity on the outcome level. By analysing cross-correlations, we highlighted patterns within social stressor and strain relations depending on the specific outcomes. Finally, we proposed that all social stressors can be integrated under the term “relational devaluation”, whereas their distinguishing features are notably due to characteristics such as frequency/duration and power differential. Obviously, there often are good reasons to define social stressors more specifically; however, authors should explicate whether a construct they propose represents a different core construct that is not covered by relational devaluation, or whether instead it represents the core construct of relational devaluation but assumes certain additional characteristics as defining features. An example for the latter is mobbing/bullying, which can be described as representing the core construct of relational devaluation plus additional characteristics in terms of powerful sources, high frequency, and long duration.

## Supplementary Information

**Additional file 1.** Definitions of the most frequently used concepts of social stressors [73–82].

**Additional file 2.** Search strategy for Ebscohost

**Additional file 3.** Flow Chart for literature search

**Additional file 4.** PICOS Framework

**Additional file 5.** References included in analysis

## Data Availability

The data sets used and/or analysed during the current study are available from the corresponding author on reasonable request.

## Abbreviations

CI: Confidence interval
CMA: Comprehensive Meta-Analysis
CWB: Counterproductive work behaviour
FTE: Full-time equivalent
FWER: Family-wise error rate
k: Amount of studies
N: Sample size
OCB: Organizational citizenship behaviour
SD: Standard deviation
SOS-model: Stress as offense to self-model
