# Supplementary material for: How are social stressors at work related to well-being and health? A systematic review and meta-analysis
Source: BMC Public Health. 2021 May 10;21:890. doi: 10.1186/s12889-021-10894-7 (PMC8111761; doi:10.1186/s12889-021-10894-7)
Supplement: Supplementary file 1 — Additional file 1. Definitions of the most frequently used concepts of social stressors [73–82]. [file 12889_2021_10894_MOESM1_ESM.docx]

**Additional file 1.** Definitions of the most frequently used concepts of social stressors

*Abusive supervision.* Describes subordinates’ perception of “the extent to which supervisors engage in the sustained display of hostile verbal and nonverbal behaviours, excluding physical contact” [71, p. 178].

*Bullying (*also described as *mobbing).* “Bullying at work means harassing, offending, or socially excluding someone or negatively affecting someone’s work. In order for the label *bullying* (or *mobbing*) to be applied to a particular activity, interaction, or process, the bullying behaviour has to occur repeatedly and regularly (e.g. weekly) and over a period of time (e.g. about six months). Bullying is an escalating process in the course of which the person confronted ends up in an inferior position and becomes the target of systematic negative social acts. A conflict cannot be called bullying if the incident is an isolated event or if two parties of approximately equal strength are in conflict” [67, p. 22].

*Discrimination.* Consists of the differential treatment of people based on actual or perceived membership in a particular group [72].

*Emotional abuse.* Interactions “characterised by hostile verbal and non-verbal, non-physical behaviours directed by one or more persons towards another that negatively affect the target’s sense of him/herself as a competent person and worker” [73, p. 176].

*Harassment.* Repeated and persistent attempts by a person to torment, wear down, frustrate, or provoke a reaction from another person; it is treatment which persistently pressures, frightens, provokes, intimidates or otherwise causes discomfort in another person [74].

*Hostility.* Being exposed to anger and disgust [75].

*Illegitimate tasks.* Illegitimate tasks violate the line between what an employee believes falls within his or her work role and what does not. By nature, they send an underlying signal of disrespect or disregard. Illegitimate task requests are considered a form of unfair treatment. The very core of what makes an illegitimate task troubling for employees is the fact that these tasks imply social signals of disrespect, devaluation, thoughtlessness, carelessness, or disregard [4, 31].

*Interpersonal conflicts.* Interpersonal conflicts in the workplace may range from minor disagreements between co-workers to physical assaults on others. The conflict may be overt (e.g. being rude to a co-worker) or covert (e.g. spreading rumours about a co-worker) [21].

*Interpersonal unfairness.* Refers to a lack of propriety, dignity, and respect from authority figures who are charged with implementing company procedures, making decisions, and distributing outcomes [76].

*Mobbing.* See bullying.

*Role ambiguity.* Uncertainty about tasks and expectation of others [1].

*Role conflict.* Incompatible demands placed upon an employee such that compliance with both would be difficult [1].

*Social exclusion.* The perception of being excluded, rejected, or ignored by others at the workplace, thus hindering one’s ability to establish or maintain positive interpersonal relationships [77].

*Undermining.* Involves “behaviors intended to hinder, over time, the ability to establish and maintain positive interpersonal relationships, work-related success, and favorable reputation” [78, p. 332].

*Victimisation.* Includes “an individual’s perception of having been exposed, either momentarily or repeatedly, to the aggressive acts of one or more other persons” [79, p. 260].

*Workplace aggression.* Refers to “a general term encompassing all forms of behaviour by which individuals attempt to harm others at work or their organisations” [80, p. 393].

*Workplace violence.* “Refers only to instances involving direct physical assaults” [80, p. 393].
