# Supplementary material for: How are social stressors at work related to well-being and health? A systematic review and meta-analysis
Source: BMC Public Health. 2021 May 10;21:890. doi: 10.1186/s12889-021-10894-7 (PMC8111761; doi:10.1186/s12889-021-10894-7)
Supplement: Supplementary file 2 — Additional file 2. Search strategy for Ebscohost [file 12889_2021_10894_MOESM2_ESM.docx]

**Search strategy for Ebscohost**

**ABSTRACT:**

(social stress* OR interpersonal* stress* OR zwischenmenschlich* Stress* OR interpersonal* conflict* OR zwischenmenschlich* Konflikt OR supervisor abuse OR missbräuchliches Vorgesetztenverhalten OR abusive supervis* OR Missbrauch Vorgesetzt* OR destructive leadership OR destruktiv* Führung* OR mobbing OR bullying OR bossing OR social undermining OR sozial* Untergraben OR sexual harassment OR sexueller Missbrauch OR harass* OR Missbrauch OR workplace revenge OR Rache OR workplace incivility OR Unhöflich* OR social incivility OR sozial* unhöflich* OR uncivil* OR rudeness OR Grobheit OR social hassle* OR sozial* schikan* OR gossip* OR läster* OR rumor OR Gerüchte OR mistreatment OR fehlbehand* OR antisocial behavior OR antisoz* Verhalten OR misbehavior OR Fehlverhalten OR aggressi* OR violence OR gewalt* OR organizational justice OR organizational* gerecht* OR procedural justice OR prozedural* Gerecht* OR interactional justice OR interactional* Gerecht* OR interpersonal justice OR zwischenmenschlich* Gerecht* OR interpersonell Gerecht* OR informational justice OR informational* Gerecht* OR justice OR Gerechtigkeit OR Fairness OR workplace abuse OR Arbeitsplatzmissbrauch OR emotional abuse OR emotional* Missbrauch* OR abuse OR Missbrauch OR verbal abuse OR verbal* Missbrauch OR micro politics OR Mikropolitik OR organizational politics OR Organisationspolitik OR offence OR Vergehen OR Delikt OR Verstoss OR Beleidi* OR Angriff OR offending OR insult* OR kränken OR beschimpfen OR ridicule* OR verspott* OR verhöhn* OR Spott OR Hohn OR tease* OR teasi* OR hänseln OR reizen OR stichel* OR necken OR piesacken OR disrespect OR respektlos* OR threat OR Angriff OR bedroh* OR hostility OR Feindselig* OR belittl* OR disparage* OR herabsetz* OR veracht* OR verumglimpf* OR demean* OR ernidrig* OR isolation OR exclusion OR Ausschluss OR ausschliessen* OR obstruction OR verhinder* OR manipulation OR interpersonal treatment OR interpersonal* Umgang OR interpersonal* Behand* OR team stress* OR relationship stress* OR Beziehungsstress* OR negative* feedback OR destructive feedback OR destruktiv* feedback OR dysfunctional feedback OR disfunktional* feedback OR abusive feedback OR missbräuchl* feedback OR hurtful feedback OR verletzend* feedback OR negative criticism OR negative* Kritik OR destructive criticism OR destruktiv* Kritik OR dysfunctional criticism OR disfunktion* Kritik OR abusive criticism OR missbräuchl* Kritik OR hurtful criticism OR verletzl* Kritik OR ostracism OR ächten OR Ächtung OR ausgrenz* OR interperson* constraint*)

**TITLE:**

AND (work* OR arbeit* OR organization OR organization* OR job* OR Beruf* OR occupation* OR Anstellung OR vocation* OR Beschäftigung OR company OR employe*)

**ABSTRACT:**

AND (absenteeism* OR Absentismus OR absence* OR Absenz*) OR (intent* to leave OR Kündigungsabsicht OR intent* to quit) OR (counterproductive work behavior OR CWB OR kontraproduktiv* Arbeitsverhalten) OR (contextual performance OR kontextuell* Leistung OR extra-role performance OR extra-role behavior OR extra-rollen Verhalten OR extra-rollen Leistung) OR (commit*) OR (performance OR Leistung OR leisten OR in-role performance OR Rollenverhalten) OR (engagement) OR motivation OR (withdrawal OR Rückzug OR rückzieh* Verhalten) OR (aggress* behavior OR aggress* Verhalten) OR (organizational citizenship behavior OR organizational* Bürgersinn OR OCB) OR (devian* Verhalten OR devian* behavior) OR (search* behavior OR Suchverhalten) OR (attendance motivation) OR (turnover* OR Kündigung OR turnover behavior) OR (innovative behavior OR innovativ* Verhalten) OR (involvement) OR intention to remain OR (antisocial behavior OR antisoz* Verhalten) OR (trait anger OR Ärger*) OR (safety compliance OR Sicherheitsverhalten) OR (prosocial behavior OR prosoz* Verhalten OR altruism*) OR (accident* OR unfall) OR (incivility OR incivil* behavior OR unhöflich*) OR (violence OR violent behavior OR gewalt) OR (presenteeism* OR presentism OR Präsentismus) OR intention to retire OR (suicide* OR Suizid*) OR ((alcohol* OR drug* OR drogen* OR smok* OR rauche*) AND (consumption OR Konsum OR abuse OR Missbrauch OR Sucht)) OR (active* OR aktiv* OR exercise* OR beweg*) OR (theft OR Diebstahl OR stehlen) OR (consumption OR Konsum*) OR (career withdrawal OR Karriererückzug) OR (tardiness OR unpünktlich*) OR (production deviance OR Devianz OR Zerstörung OR property deviance OR Eigentumszerstörung OR Eigentumsdevianz OR political deviance OR politisch* Devianz OR personal aggression OR persönl* Aggression OR stealing OR sabotage) OR (help* OR Hilfesuche OR Hilfeverhalten) OR (self-destruct* behavior OR Selbstzerstörung OR selbstzerstör* Verhalten) OR (adaption OR adaptive behavior OR anpassend* Verhalten) OR (career behavior OR Karriereverhalten) OR (proactive behavior OR proaktiv* Verhalten) OR (efficiency OR Effizienz OR effizient* behavior)

**TITLE & ABSTRACT:**

NOT (military OR Militär OR climat* OR Klima OR unemploy* OR arbeitslos* OR erwerbslos* OR animal OR tier* OR psychotherapy* OR psychiatric* OR psychotherapie OR peer OR adolescent* OR jugendl* OR sex work* OR Sexarbeit OR Front OR war OR Krieg* OR veteran OR soldier OR Soldat OR neuro* OR care OR therap* OR EEG OR migration* OR bio* OR chemi* OR Experiment* OR labor* OR Cortex OR cranial OR rat* OR mice OR mouse OR Maus OR Affe OR Monkey OR Gen* OR metabolis* OR molecular* OR molekul* OR klon* OR cell* OR photo* OR racism OR frog OR autism OR down* syndrome OR psychoactive* OR lymph* OR hybrid OR bible OR fish OR parasitic OR pollution OR polymer* OR pesticide OR emission OR nucl* OR robot* OR terror* OR wasp* OR sinus OR membrane* OR phylo* OR binge* OR fulva OR pirat* OR fiction* OR comment* OR game theor* OR HIV OR infravesical OR “domestic abuse” OR “domestic violenc*” OR “child abuse” OR “spouse abuse” OR “spouse violence” OR “partner abuse” OR “partner violence” OR Wlan OR wireless OR microwave OR vibration)
