# Supplementary material for: How are social stressors at work related to well-being and health? A systematic review and meta-analysis
Source: BMC Public Health. 2021 May 10;21:890. doi: 10.1186/s12889-021-10894-7 (PMC8111761; doi:10.1186/s12889-021-10894-7)
Supplement: Supplementary file 3 — Additional file 3. Flow Chart for literature search [file 12889_2021_10894_MOESM3_ESM.doc]

**Supplement 2. Flow chart for literature search according to the PRISMA statement**

**Screening**

**Included**

**Eligibility**

**Identification**

Records identified through database searching within 3 waves
(n = 56.497)

Additional records identified through other sources
(n = 37)

Records after duplicates removed
(n = 42.147)

Records screened
(n = 42.147)

Records excluded based on title and abstract screening (n = 40.150)

Full-text articles assessed for eligibility
(n = 1.997)

Full-text articles excluded
(n = 1.290)

Studies included in database
(n = 707)

Studies included in quantitative synthesis (meta-analysis)
(n = 557)
