# Supplementary material for: How are social stressors at work related to well-being and health? A systematic review and meta-analysis
Source: BMC Public Health. 2021 May 10;21:890. doi: 10.1186/s12889-021-10894-7 (PMC8111761; doi:10.1186/s12889-021-10894-7)
Supplement: Supplementary file 4 — Additional file 4. PICOS Framework [file 12889_2021_10894_MOESM4_ESM.docx]

**Supplement 4. PICOS Framework for outlining the components of the research question**

| PICOS element | Definition | Inclusion/exclusion criteria |
| --- | --- | --- |
| Population | Working population with social contact at work. | Adults working 50% or more of a full-time equivalent (FTE). Student samples were included if they worked at least 50% of a FTE.  We excluded military workers, children, non-working adults and retired persons, as well as clinical samples. |
| Outcomes | Differences or communalities in the relationships between different social stressors at work and well-being- and health-related outcomes represented by a correlation. | Social stressors: role stress, interpersonal conflicts (supervisor, clients), perceived victimization, supervisor mistreatment, mobbing/bullying, social exclusion, sexual mistreatment, harassment, incivility, identity threat, physical violence, verbal/emotional violence, mistreatment, stereotype threat, hostility, undermining, illegitimate tasks, lack of justice  Outcomes:   - well-being/health (high and low arousal, physical, mental, general, burnout) - behaviour (turnover intention, absenteeism, OCB, performance, CWB) - attitudes (commitment, life satisfaction, job satisfaction) |
| Study design | - Cross-sectional - Longitudinal - Prospective - Quantitative | All study designs could be included if they reported a quantitatively measured correlation as described in “Outcomes”. |
| Additional elements | Characteristics of reports | - Written in English or German - Contains quantitative data - Only published research included to ensure decent quality |

*Note.* The elements “Intervention” and “Comparison” are not applicable to our research project.
