# Supplementary material for: How are social stressors at work related to well-being and health? A systematic review and meta-analysis
Source: BMC Public Health. 2021 May 10;21:890. doi: 10.1186/s12889-021-10894-7 (PMC8111761; doi:10.1186/s12889-021-10894-7)
Supplement: Supplementary file 5 — Additional file 5. References included in analysis [file 12889_2021_10894_MOESM5_ESM.docx]

**Reference list of studies included in analysis**

1. Acker GM. An examination of the relationships between workers and agencies characteristics and three outcome variables: Burnout, role stress, and intent to quit. Am J Psychiatr Rehabil. 2008;11:295-309.
2. Adams GA, Buck J. Social stressors and strain among police officers: It’s not just the bad guys. Crim Justice Behav. 2010;37:1030-40.
3. Addae HM, Praveen Parboteeah K, Velinor N. Role stressors and organizational commitment: Public sector employment in St Lucia. Int J Manpow. 2008;29:567-82.
4. Adebayo DO, Sunmola AM, Udegbe, IB. Workplace fairness and emotional exhaustion in Nigeria police: The moderating role of gender. Anxiety Stress Coping. 2008;21:405-16.
5. Ali N, Jan S. Relationship between organizational justice and organizational commitment and turnover intentions amongst medical representatives of pharmaceuticals companies of Pakistan. J Manag Sci. 2012;6:201-12.
6. Almost J, Doran DM, McGillis Hall L, Spence Laschinger HK. Antecedents and consequences of intra-group conflict among nurses. J Nurs Manag. 2010;18:981-92.
7. Alotaibi AG. Antecedents of organizational citizenship behavior: A study of public personnel in Kuwait. Public Pers Manag. 2001;30:363-76.
8. Andrews MC, Kacmar KM, Harris KJ. Got political skill? The impact of justice on the importance of political skill for job performance. J Appl Psychol. 2009;94: 1427-37.
9. Anjum MA, Parvez A. Counterproductive behavior at work: A comparison of blue collar and white collar workers. Pak J Commer Soc Sci 2013;7:417-34.
10. Appelberg K, Romanov K, Honkasalo, M-L, Koskenvuo M. Interpersonal conflicts at work and psychosocial characteristics of employees. Soc Sci Med. 1991;32:1051-56.
11. Aquino K, Bommer WH. Preferential mistreatment: How victim status moderates the relationship between organizational citizenship behavior and workplace victimization. Organ Sci. 2003;14:374-85.
12. Aquino K, Griffeth RW, Allen DG, Hom PW. Integrating justice constructs into the turnover process: A test of a Referent Cognitions Model. Acad Manag J. 1997;40:1208-27.
13. Aquino K, Lewis MU, Bradfield M. Justice constructs, negative affectivity, and employee deviance: A proposed model and empirical test. J Organ Behav. 1999;20:1073-91.
14. Armstrong-Stassen M, Schlosser F. Perceived organizational membership and the retention of older workers. J Organ Behav. 2011;32:319-44.
15. Aryee S, Budhwar PS, Chen ZX. Trust as a mediator of the relationship between organizational justice and work outcomes: Test of a social exchange model. J Organ Behav.
16. Aryee S, Chen ZX, Budhwar PS. Exchange fairness and employee performance: An examination of the relationship between organizational politics and procedural justice. Organ Behav Hum Decis Process. 2004;94:1-14.
17. Aryee S, Sun L-Y, Chen ZXG, Debrah YA. Abusive supervision and contextual performance: The mediating role of emotional exhaustion and the moderating role of work unit structure. Manag Organ Rev. 2008;4:393-411.
18. Asgari A, Silong AD, Ahmad A, Samah BA. The relationship between leader-member exchange, organizational inflexibility, perceived organizational support, interactional justice and organizational citizenship behaviour. Afr J Bus Manag. 2008;2:138-45.
19. Ashforth BE. Petty tyranny in organizations: A preliminary examination of antecedents and consequences. Can J Adm Sci. 1997;14:126-40.
20. Baker TL, Hunt TG, Andrews MC. Promoting ethical behavior and organizational citizenship behaviors: The influence of corporate ethical values. J Bus Res, 2006;59:849-57.
21. Balducci C, Alfano V, Fraccaroli F. Relationships between mobbing at work and MMPI-2 personality profile, posttraumatic stress symptoms, and suicidal ideation and behavior.
22. Balducci C, Schaufeli WB, Fraccaroli F. The job demands–resources model and counterproductive work behaviour: The role of job-related affect. Eur J Work Organ Psychol, 2011;20:467-96.
23. Barclay LJ, Kiefer T. Approach or avoid? Exploring overall justice and the differential effects of positive and negative emotions. J Manag. 2014;40:1857-98.
24. Barling J, Dekker I, Loughlin CA, Kelloway EK, Fullagar C, Johnson D. Prediction and replication of the organizational and personal consequences of workplace sexual harassment. J Manag Psychol. 1996;11:4-25.
25. Barling J, Rogers AG, Kelloway EK. Behind closed doors: In-home workers’ experience of sexual harassment and workplace violence. J Occup Health Psychol. 2001;6:255-69.
26. Barton LC, Ambrosini V. The moderating effect of organizational change cynicism on middle manager strategy commitment. Int J Hum Resour Manag. 2013;24:721-46.
27. Baruch Y. Bullying on the net: Adverse behavior on e-mail and its impact. Inf Manag. 2005;42:361-71.
28. Beattie L, Griffin B. Day-level fluctuations in stress and engagement in response to workplace incivility: A diary study. Work Stress, 2014;28:124-42.
29. Beauregard TA. Fairness perceptions of work−life balance initiatives: Effects on counterproductive work behaviour. Br J Manag. 2014;25:772-89.
30. Beehr TA, Drexler Jr JA, Faulkner S. Working in small family businesses: Empirical comparisons to non-family businesses. J Organ Behav. 1997;18:297-312.
31. Begley TM, Lee C, Fang Y, Li J. Power distance as a moderator of the relationship between justice and employee outcomes in a sample of Chinese employees. J Manag Psychol. 2002;17:692-711.
32. Begley TM, Lee C, Hui C. Organizational level as a moderator of the relationship between justice perceptions and work-related reactions. J Organ Behav. 2006;27:705-21.
33. Behson SJ. Which dominates? The relative importance of work–family organizational support and general organizational context on employee outcomes. J Vocat Behav. 2002;61:53-72.
34. Berdahl JL, Aquino K. Sexual behavior at work: Fun or folly? J Appl Psychol. 2009;94:34-47.
35. Bettencourt LA, Brown SW. Contact employees: Relationships among workplace fairness, job satisfaction and prosocial service behaviors. J Retail. 1997;73:39-61.
36. Birkeland Nielsen M, Hetland J, Matthiesen SB, Einarsen S. Longitudinal relationships between workplace bullying and psychological distress. Scand J Work Environ Health. 2012;38:38-46.
37. Blau G, Andersson L. Testing a measure of instigated workplace incivility. J Occup Organ Psychol. 2005;78:595-614.
38. Boddy CR. Corporate psychopaths, conflict, employee affective well-being and counterproductive work behaviour. J Bus Ethics. 2014;121:107-21.
39. Bond MA, Punnett L, Pyle JL, Cazeca D, Cooperman M. Gendered work conditions, health, and work outcomes. J Occup Health Psychol. 2004;9:28-45.
40. Borg MG, Riding RJ, Falzon JM. Stress in teaching: A study of occupational stress and its determinants, job satisfaction and career commitment among primary schoolteachers. Educ Psychol. 1991;11:59-75.
41. Botsford Morgan W, King EB. Mothers’ psychological contracts: Does supervisor breach explain intention to leave the organization? Hum Resour Manag. 2012;51:629-49.
42. Bowler WM, Brass DJ. Relational correlates of interpersonal citizenship behavior: A social network perspective. J Appl Psychol. 2006;91:70-82.
43. Bowling NA, Eschleman KJ. Employee personality as a moderator of the relationships between work stressors and counterproductive work behavior. J Occup Health Psychol. 2010;15:91-103.
44. Boz M, Martínez I, Munduate L. Breaking negative consequences of relationship conflicts at work: The moderating role of work family enrichment and supervisor support. J Work Organ Psychol. 2009;25:113-21.
45. Brashear TG, Brooks CM, Boles JS. Distributive and procedural justice in a sales force context: Scale development and validation. J Bus Res. 2004;57:86-93.
46. Brashear TG, Manolis C, Brooks CM. The effects of control, trust, and justice on salesperson turnover. J Bus Res. 2005;58:241-9.
47. Brennan A, Skarlicki DP. Personality and perceived justice as predictors of survivors’ reactions following downsizing. J Appl Soc Psychol. 2004;34:1306-28.
48. Brotheridge CM, Lee RT. Restless and confused: Emotional responses to workplace bullying in men and women. Career Dev Int. 2010;15:687-707.
49. Brough P. Workplace violence experienced by paramedics: Relationships with social support, job satisfaction, and psychological strain. Australas J Disaster Trauma Stud. 2005;2:1-12.
50. Brown G, Bemmels B, Barclay LJ. The importance of policy in perceptions of organizational justice. Hum Relat. 2010;63:1587-609.
51. Brown ME, Treviño LK, Harrison DA. Ethical leadership: A social learning perspective for construct development and testing. Organ Behav Hum Decis Process. 2005;97:117-34.
52. Brown Johnson N, Jarley P. Justice and union participation: An extension and test of mobilization theory. Br J Ind Relat. 2004;42:543-62.
53. Bruk-Lee V, Nixon AE, Spector PE. An expanded typology of conflict at work: Task, relationship and non-task organizational conflict as social stressors. Work Stress.
54. Bruk-Lee V, Spector PE. The social stressors–counterproductive work behaviors link: Are conflicts with supervisors and coworkers the same? J Occup Health Psychol. 2006;11:145-56.
55. Budd JW, Arvey RD, Lawless P. Correlates and consequences of workplace violence. J Occup Health Psychol. 1996;1:197-210.
56. Buluc B, Gunes AM. Relationship between organizational justice and organizational commitment in primary schools. Anthropol. 2014;18:145-52.
57. Büssing A, Höge T. Aggression and violence against home care workers. J Occup Health Psychol. 2004;9:206-19.
58. Buttigieg SC, West MA, Dawson JF. Well-structured teams and the buffering of hospital employees from stress. Health Serv Manag Res. 2011;24:203-12.
59. Campbell NS, Perry SJ, Maertz Jr CP, Allen DG, Griffeth RW. All you need is ... resources: The effects of justice and support on burnout and turnover. Hum Relat. 2013;66:759-82.
60. Casimir G, McCormack D, Djurkovic N, Nsubuga-Kyobe A. Psychosomatic model of workplace bullying: Australian and Ugandan schoolteachers. Empl Relat. 2012;34:411-28.
61. Chan D. Interactive effects of situational judgment effectiveness and proactive personality on work perceptions and work outcomes. J Appl Psychol. 2006;91:475-81.
62. Chang E. Employees’ overall perception of HRM effectiveness. Hum Relat. 2005;58:523-44.
63. Chen H, Jin Y-H. The effects of organizational justice on organizational citizenship behavior in the Chinese context: The mediating effects of social exchange relationship. Public Pers Manag. 2014;43:301-13.
64. Chen M-F, Lin C-P, Lien G-Y. Modelling job stress as a mediating role in predicting turnover intention. Serv Ind J. 2011;31:1327-45.
65. Chen PY, Spector PE. Negative affectivity as the underlying cause of correlations between stressors and strains. J Appl Psychol. 1991;76:398-407.
66. Chiaburu DS, Lim AS. Manager trustworthiness or interactional justice? Predicting organizational citizenship behaviors. J Bus Ethics. 2008;83:453-67.
67. Chien MS, Lawler JS, Uen J-F. Performance-based pay, procedural justice and job performance for R&D professionals: Evidence from the Taiwanese high-tech sector. Int J Hum Resour Manag. 2010;21:2234-48.
68. Chou T-Y, Chou S-CT, Jiang JJ, Klein G. The organizational citizenship behavior of IS personnel: Does organizational justice matter? Inf Manag. 2013;50:105-11.
69. Choudhary N, Deswal RK, Philip PJ. Impact of organizational justice on employees’ workplace and personal outcomes: A study of Indian insurance sector. IUP J Organ Behav. 2013;12:7-20.
70. Chu C-I, Lee M-S, Hsu H-M. The impact of social support and job stress on public health nurses’ organizational citizenship behaviors in rural Taiwan. Public Health Nurs. 2006;23:496-505.
71. Claybourn M. Relationships between moral disengagement, work characteristics and workplace harassment. J Bus Ethics. 2011;100:283-301.
72. Cohen-Charash Y, Mueller JS. Does perceived unfairness exacerbate or mitigate interpersonal counterproductive work behaviors related to envy? J Appl Psychol. 2007;92:666-80.
73. Collins BJ, Mossholder KW, Taylor SG. Does process fairness affect job performance? It only matters if they plan to stay. J Organ Behav. 2012;33:1007-26.
74. Colquitt JA, LePine JA, Piccolo RF, Zapata CP, Rich BL. Explaining the justice–performance relationship: Trust as exchange deepener or trust as uncertainty reducer? J Appl Psychol. 2012;97:1-15.
75. Cortina LM. Hispanic perspectives on sexual harassment and social support. Pers Soc Psychol Bull. 2004;30:570-84.
76. Cortina LM, Fitzgerald LF, Drasgow F. Contextualizing Latina experiences of sexual harassment: Preliminary tests of a structural model. Basic and Applied Social Psychology, 2002;24:295-311.
77. Cortina LM, Magley VJ. Raising voice, risking retaliation: Events following interpersonal mistreatment in the workplace. J Occup Health Psychol. 2003;8:247-65.
78. Crede M, Chernyshenko OS, Stark S, Dalal RS, Bashshur M. Job satisfaction as mediator: An assessment of job satisfaction’s position within the nomological network. J Occup Organ Psychol. 2007;80:515-38.
79. Cubela Adoric V, Kvartuc T. Effects of mobbing on justice beliefs and adjustment. Eur Psychol. 2007;12:261-71.
80. Dai Y-D, Dai Y-Y, Chen K-Y, Wu H-C. Transformational vs transactional leadership: Which is better? A study on employees of international tourist hotels in Taipei city. Int J Contemp Hosp Manag. 2013;25:760-78.
81. Dailey RC, Kirk DJ. Distributive and procedural justice as antecedents of job dissatisfaction and intent to turnover. Hum Relat. 1992;45:305-17.
82. Daly JP, Geyer PD. The role of fairness in implementing large-scale change: Employee evaluations of process and outcome in seven facility relocations. J Organ Behav. 1994;15:623-38.
83. Dasgupta SA, Suar D, Singh S. Impact of managerial communication styles on employees’ attitudes and behaviours. Empl Relat. 2013;35:173-99.
84. De Cremer D, Brockner J, Fishman A, Van Dijke M, van Olffen W, Mayer DM. When do procedural fairness and outcome fairness interact to influence employees’ work attitudes and behaviors? The moderating effect of uncertainty. J Appl Psychol. 2010;95:291-304.
85. De Gieter S, De Cooman R, Hofmans J, Pepermans R, Jegers M. Pay-level satisfaction and psychological reward satisfaction as mediators of the organizational justice–turnover intention relationship. Int Stud Manag Organ. 2012;42:50-67.
86. De Jong J, Schalk R. Extrinsic motives as moderators in the relationship between fairness and work-related outcomes among temporary workers. J Bus Psychol. 2010;25:175-89.
87. De Pedro MM, Sánchez MIS, Navarro MCS, Izquierdo MG. Workplace mobbing and effects on workers’ health. Span J Psychol. 2008;11:219-27.
88. DeConinck JB, Johnson JT. The effects of perceived supervisor support, perceived organizational support, and organizational justice on turnover among salespeople. J Pers Sell Sales Manag. 2009;29:333-50.
89. Decoster S, Camps J, Stouten J. The mediating role of LMX between abusive supervision and work behaviors: A replication and extension. Am J Bus. 2014;29:61-75.
90. Deery S, Walsh J, Guest D. Workplace aggression: The effects of harassment on job burnout and turnover intentions. Work Employ Soc. 2011;25:742-59.
91. Deery S, Walsh J, Zatzick CD. A moderated mediation analysis of job demands, presenteeism, and absenteeism. J Occup Organ Psychol. 2014;87:352-69.
92. Detert JR, Treviño LK, Burris ER, Andiappan M. Managerial modes of influence and counterproductivity in organizations: A longitudinal business-unit-level investigation. J Appl Psychol. 2007;92: 993-1005.
93. Devonish D, Greenidge D. The effect of organizational justice on contextual performance, counterproductive work behaviors, and task performance: Investigating the moderating role of ability‐based emotional intelligence. Int J Sel Assess. 2010;18:75-86.
94. Díaz-Gracia L, Barbaranelli C, Moreno-Jiménez B. Spanish version of Colquitt’s Organizational Justice Scale. Psicothema, 2014;26:538-44.
95. Diekmann KA, Barsness ZI, Sondak H. Uncertainty, fairness perceptions, and job satisfaction: A field study. Soc Justice Res. 2004;17:237-55.
96. Dijkstra MTM, Beersma B, Evers A. Reducing conflict-related employee strain: The benefits of an internal locus of control and a problem-solving conflict management strategy. Work Stress. 2011;25:167-84.
97. Dijkstra MTM, De Dreu CKW, Evers A, van Dierendonck D. Passive responses to interpersonal conflict at work amplify employee strain. Eur J Work Organ Psychol. 2009;18:405-23.
98. Dijkstra MTM, van Dierendonck D, Evers A. Responding to conflict at work and individual well-being: The mediating role of flight behaviour and feelings of helplessness. Eur J Work Organ Psychol. 2005;14:119-35.
99. Dijkstra MTM, van Dierendonck D, Evers A, De Dreu CKW. Conflict and well-being at work: The moderating role of personality. J Manag Psychol. 2005;20:87-104.
100. Dionisi AM, Barling J, Dupré KE. Revisiting the comparative outcomes of workplace aggression and sexual harassment. J Occup Health Psychol. 2012;17:398-408.
101. Djurkovic N, McCormack D, Casimir G. The physical and psychological effects of workplace bullying and their relationship to intention to leave: A test of the psychosomatic and disability hypotheses. Int J Organ Theory Behav. 2004;7:469-97.
102. Djurkovic N, McCormack D, Casimir G. Neuroticism and the psychosomatic model of workplace bullying. J Manag Psychol. 2006;21:73-88.
103. Djurkovic N, McCormack D, Casimir G. Workplace bullying and intention to leave: The moderating effect of perceived organisational support. Hum Resour Manag J. 2008;18:405-22.
104. Donovan MA, Drasgow F, Munson LJ. The Perceptions of Fair Interpersonal Treatment Scale: Development and validation of a measure of interpersonal treatment in the workplace. J Appl Psychol. 1998;83: 683-92.
105. Dormann C, Zapf D. Social support, social stressors at work, and depressive symptoms: Testing for main and moderating effects with structural equations in a three-wave longitudinal study. J Appl Psychol. 1999;84: 874-84.
106. Dormann C, Zapf D. Customer-related social stressors and burnout. J Occup Health Psychol. 2004;9:61-82.
107. Dormann C, Zapf D, Isic A. Emotionale Arbeitsanforderungen und ihre Konsequenzen bei Call Center-Arbeitsplätzen [Emotional requirements at work and their consequences for call-center jobs]. Z Arb Organ. 2002;46:201-15.
108. Doucet L. Service provider hostility and service quality. Acad Manag J. 2004;47:761-71.
109. Duffy MK, Ganster DC, Pagon M. Social undermining in the workplace. Acad Manag J. 2002;45:331-51.
110. Duffy MK, Ganster DC, Shaw JD, Johnson JL, Pagon M. The social context of undermining behavior at work. Organ Behav Hum Decis Process. 2006;101:105-26.
111. Eberz S, Becker R, Antoni CH. Kohärenzerleben im Arbeitskontext: Ein nützliches Konstrukt für die ABO-Psychologie? [Work-related sense of coherence: A useful construct for occupational psychology?]. Z Arb Organ. 2011;55:115-31.
112. Edwards JA, Guppy A, Cockerton T. A longitudinal study exploring the relationships between occupational stressors, non-work stressors, and work performance. Work Stress. 2007;21:99-116.
113. Einarsen S, Hoel H, Notelaers, G. Measuring exposure to bullying and harassment at work: Validity, factor structure and psychometric properties of the Negative Acts Questionnaire-Revised. Work Stress. 2009;23:24-44.
114. Einarsen S, Raknes BI. Harassment in the workplace and the victimization of men. Violence Vict. 1997;12:247-63.
115. Einarsen S, Raknes BI, Matthiesen SB. Bullying and harassment at work and their relationships to work environment quality: An exploratory study. European Work and Organizational Psychologist, Eur Work Organ Psychol. 1994;4:381-401.
116. Elçi M, Karabay ME, Alpkan L, Şener İ. The mediating role of mobbing on the relationship between organizational silence and turnover intention. Procedia Soc Behav Sci. 2014;150:1298-309.
117. Elovainio M, van den Bos K, Linna A, Kivimäki M, Ala-Mursula L, Pentti J, Vahtera J. Combined effects of uncertainty and organizational justice on employee health: Testing the Uncertainty Management Model of Fairness Judgments among Finish public sector employees. Soc Sci Med. 2005;61:2501-12.
118. Evers W, Tomic W, Brouwers A. Aggressive behaviour and burnout among staff of homes for the elderly. Int J Ment Health Nurs. 2002;11:2-9.
119. Fang Y-H, Chiu C-M. In justice we trust: Exploring knowledge-sharing continuance intentions in virtual communities of practice. Comput Human Behav. 2010;26:235-46.
120. Farh CIC, Chen Z. Beyond the individual victim: Multilevel consequences of abusive supervision in teams. J Appl Psychol. 2014;99:1074-95.
121. Farh J-L, Podsakoff PM, Organ DW. Accounting for organizational citizenship behavior: Leader fairness and task scope versus satisfaction. J Manag. 1990;16:705-21.
122. Farmer SJ, Beehr TA, Love KG. Becoming an undercover police officer: A note on fairness perceptions, behavior, and attitudes. J Organ Behav. 2003;24:373-87.
123. Ferris DL, Brown DJ, Berry JW, Lian H. The development and validation of the Workplace Ostracism Scale. J Appl Psychol. 2008;93:1348-66.
124. Ferris DL, Spence JR, Brown DJ, Heller D. Interpersonal injustice and workplace deviance: The role of esteem threat. J Manag. 2012;38:1788-811.
125. Figueiredo‐Ferraz H, Gil‐Monte PR, Grau‐Alberola E, Llorca‐Pellicer M, García‐Juesas JA. Influence of some psychosocial factors on mobbing and its consequences among employees working with people with intellectual disabilities. J Appl Res Intellect Disabil. 2012;25:455-63.
126. Finne LB, Knardahl S, Lau B. Workplace bullying and mental distress – a prospective study of Norwegian employees. Scand J Work Environ Health. 2011;37:276-87.
127. Firth-Cozens J. Sources of stress in women junior house officers. Br Med J. 1990;301:89-91.
128. Fitzgerald LF, Drasgow F, Hulin CL, Gelfand MJ, Magley VJ. Antecedents and consequences of sexual harassment in organizations: A test of an integrated model. J Appl Psychol. 1997;82:578-89.
129. Flint D, Haley LM, McNally JJ. Individual and organizational determinants of turnover intent. Pers Review. 2013;42:552-72.
130. Fortunato VJ. A comparison of the construct validity of three measures of negative affectivity. Educ Psychol Meas. 2004;64:271-89.
131. Fox S, Stallworth LE. Racial/ethnic bullying: Exploring links between bullying and racism in the US workplace. J Vocat Behav. 2005;66:438-56.
132. Fox S, Stallworth LE. The battered apple: An application of stressor-emotion-control/support theory to teachers’ experience of violence and bullying. Hum Relat. 2010;63:927-54.
133. Fox S, Spector PE, Goh A, Bruursema K. Does your coworker know what you’re doing? Convergence of self- and peer-reports of counterproductive work behavior. Int J Stress Manag. 2007;14:41-60.
134. Fox S, Spector PE, Miles D. Counterproductive work behavior (CWB) in response to job stressors and organizational justice: Some mediator and moderator tests for autonomy and emotions. J Vocat Behav. 2001;59:291-309.
135. Francis L, Barling J. Organizational injustice and psychological strain. Can J Behav Sci. 2005;37:250-61.
136. Frese M, Zapf D. Eine Skala zur Erfassung von sozialen Stressoren am Arbeitsplatz [A scale to measure social stressors at work]. Z Arbeitswiss. 1987;41:134-41.
137. Frone MR. Predictors of work injuries among employed adolescents. J Appl Psychol. 1998;83:565-76.
138. Frone MR. Interpersonal conflict at work and psychological outcomes: Testing a model among young workers. J Occup Health Psychol. 2000;5:246-55.
139. Fugate M, Prussia GE, Kinicki AJ. Managing employee withdrawal during organizational change: The role of threat appraisal. J Manag 2012;38:890-914.
140. Fujiwara K, Tsukishima E, Tsutsumi A, Kawakami N, Kishi R. Interpersonal conflict, social support, and burnout among home care workers in Japan. J Occup Health. 2003;45:313-20.
141. Gabler CB, Nagy KR, Hill RP. Causes and consequences of abusive supervision in sales management: A tale of two perspectives. Psychol Mark. 2014;31:278-93.
142. Gabris GT, Ihrke DM. Does performance appraisal contribute to heightened levels of employee burnout? The results of one study. Public Pers Manag. 2001;30:157-72.
143. Gao RG, Wang BY. Loyalty to supervisor mediated the relationship between abusive supervision and loyalty to organization. In: Liu X, Zhang H, Ruimei Z, editors. Proceedings of 2010 international conference on management science and engineering. Marrickville: Orient Academic Forum; 2010. p. 504-10.
144. Garrosa E, Rainho C, Moreno-Jiménez B, Monteiro MJ. The relationship between job stressors, hardy personality, coping resources and burnout in a sample of nurses: A correlational study at two time points. Int J Nurs Stud. 2010;47:205-15.
145. Gazica M, Meier LL, Spector PE. (2012, October 30 - November 3). Abuse and interpersonal misbehavior in public school teachers. Paper presented at the 11th Annual Meeting of the Southern Management Association, Ft. Lauderdale, FL, USA.
146. Gelens J, Hofmans J, Dries N, Pepermans R. Talent management and organisational justice: Employee reactions to high potential identification. Hum Resour Manag. J. 2014;24:159-75.
147. George JM. State or trait: Effects of positive mood on prosocial behaviors at work. J Appl Psychol. 1991;76:299-307.
148. Gettman HJ, Gelfand MJ. When the customer shouldn’t be king: Antecedents and consequences of sexual harassment by clients and customers. J Appl Psychol. 2007;92:757-70.
149. Geurts S, Rutte C, Peeters M. Antecedents and consequences of work–home interference among medical residents. Soc Sci Med. 1999;48:1135-48.
150. Ghosh R, Reio Jr TG, Bang H. Reducing turnover intent: Supervisor and co-worker incivility and socialization-related learning. Hum Resour Dev Int. 2013;16:169-85.
151. Gibney R, Zagenczyk TJ, Fuller JB, Hester K, Caner T. Exploring organizational obstruction and the expanded model of organizational identification. J Appl Soc Psychol. 2011;41:1083-109.
152. Giebels E, Janssen O. Conflict stress and reduced well-being at work: The buffering effect of third-party help. Eur J Work Organ Psychol. 2005;14:137-55.
153. Gilin Oore D, Leblanc D, Day A, Leiter MP, Laschinger HKS, Price SL, Latimer M. When respect deteriorates: Incivility as a moderator of the stressor–strain relationship among hospital workers. J Nurs Manag. 2010;18:878-88.
154. Gilstrap JB, Collins BJ. The importance of being trustworthy: Trust as a mediator of the relationship between leader behaviors and employee job satisfaction. J Leadersh Organ Stud. 2012;19:152-63.
155. Giorgi G. Workplace bullying partially mediates the climate-health relationship. J Manag Psychol. 2010;25:727-40.
156. Giumetti GW, McKibben ES, Hatfield AL, Schroeder AN, Kowalski RM. Cyber incivility @ work: The new age of interpersonal deviance. Cyberpsychol Behav Soc Netw. 2012;15:148-54.
157. Glambek M, Matthiesen SB, Hetland J, Einarsen S. Workplace bullying as an antecedent to job insecurity and intention to leave: A 6‐month prospective study. Hum Resour Manag. J. 2014;24:255-68.
158. Glasø L, Bele E, Nielsen MB, Einarsen S. Bus drivers’ exposure to bullying at work: An occupation-specific approach. Scand J Psychol. 2011;52:484-93.
159. Glasø L, Notelaers G. Workplace bullying, emotions, and outcomes. Violence Vict. 2012;27:360-77.
160. Glasø L, Vie TL, Holmdal GR, Einarsen S. An application of Affective Events Theory to workplace bullying. Eur Psychol. 2011;16:198-208.
161. Glomb TM, Liao H. Interpersonal aggression in work groups: Social influence, reciprocal, and individual effects. Acad Manag J. 2003;46:486-496.
162. Glomb TM, Richman WL, Hulin CL, Drasgow F, Schneider KT, Fitzgerald LF. Ambient sexual harassment: An integrated model of antecedents and consequences. Organ Behav Hum Decis Process. 1997;71:309-28.
163. Golden TD, Veiga JF, Dino RN. The impact of professional isolation on teleworker job performance and turnover intentions: Does time spent teleworking, interacting face-to-face, or having access to communication-enhancing technology matter? J Appl Psychol. 2008;93:1412-21.
164. Goldenhar LM, Swanson NG, Hurrell Jr JJ, Ruder A, Deddens J. Stressors and adverse outcomes for female construction workers. J Occup Health Psychol. 1998;3:19-32.
165. Goldenhar LM, Williams LJ, Swanson NG. Modelling relationships between job stressors and injury and near-miss outcomes for construction labourers. Work Stress. 2003;17:218-40.
166. González-Morales MG, Rodríguez I, Peiró JM. A longitudinal study of coping and gender in a female-dominated occupation: Predicting teachers’ burnout. J Occup Health Psychol. 2010;15:29-44.
167. Goussinsky R. Does customer aggression more strongly affect happy employees? The moderating role of positive affectivity and extraversion. Motiv Emot. 2011;35:220-34.
168. Goussinsky R. The moderating role of support seeking versus perceived social support in the relationship between customer aggression and job outcomes. Int J Organ Theory Behav. 2013;16:165-92.
169. Grandey A, Foo SC, Groth M, Goodwin RE. Free to be you and me: A climate of authenticity alleviates burnout from emotional labor. J Occup Health Psychol. 2012;17:1-14.
170. Grandey AA, Dickter DN, Sin H-P. The customer is not always right: Customer aggression and emotion regulation of service employees. J Organ Behav. 2004;25:397-418.
171. Grandey AA, Kern JH, Frone MR. Verbal abuse from outsiders versus insiders: Comparing frequency, impact on emotional exhaustion, and the role of emotional labor. J Occup Health Psychol. 2007;12:63-79.
172. Grebner S, Semmer NK, Lo Faso L, Gut S, Kälin W, Elfering A. Working conditions, well-being, and job-related attitudes among call centre agents. Eur J Work Organ Psychol. 2003;12:341-65.
173. Greenidge D, Coyne I. Job stressors and voluntary work behaviours: Mediating effect of emotion and moderating roles of personality and emotional intelligence. Hum Resour Manag. J. 2014;24:479-95.
174. Gregory BT, Osmonbekov T, Gregory ST, Albritton MD, Carr JC. Abusive supervision and citizenship behaviors: Exploring boundary conditions. J Manag Psychol. 2013;28:628-44.
175. Guerra JM, Martínez I, Munduate L, Medina FJ. A contingency perspective on the study of the consequences of conflict types: The role of organizational culture. Eur J Work Organ Psychol. 2005;14:157-76.
176. Guidroz AM, Burnfield-Geimer JL, Clark O, Schwetschenau HM, Jex SM. The Nursing Incivility Scale: Development and validation of an occupation-specific measure. J Nurs Meas. 2010;18:176-200.
177. Guidroz AM, Wang M, Perez LM. Developing a model of source-specific interpersonal conflict in health care. Stress Health. 2011;28:69-79.
178. Gumusluoglu L, Karakitapoğlu-Aygün Z, Hirst G. Transformational leadership and R&D workers’ multiple commitments: Do justice and span of control matter? J Bus Res. 2013;66:2269-78.
179. Gupta V, Singh S. An empirical study of the dimensionality of organizational justice and its relationship with organizational citizenship behaviour in the Indian context. Int J Hum Resour Manag. 2013;24:1277-99.
180. Hamaideh SH. Occupational stress, social support, and quality of life among Jordanian mental health nurses. Issues Ment Health Nurs. 2012;33:15-23.
181. Hang-yue N, Foley S, Loi R. Work role stressors and turnover intentions: A study of professional clergy in Hong Kong. Int J Hum Resour Manag. 2005;16:2133-46.
182. Hansen AM, Byrne ZS, Kiersch CE. Development and validation of an abridged measure of organizational justice. J Psychol. 2013;147:217-44.
183. Hansen ÅM, Hogh A, Persson R. Frequency of bullying at work, physiological response, and mental health. J Psychosom Res. 2011;70:19-27.
184. Hansen ÅM, Hogh A, Persson R, Karlson B, Garde AH, Ørbæk P. Bullying at work, health outcomes, and physiological stress response. J Psychosom Res. 2006;60:63-72.
185. Haq IU. The impact of interpersonal conflict on job outcomes: Mediating role of perception of organizational politics. Procedia Soc Behav Sci. 2011;25:287-310.
186. Harlos KP, Axelrod LJ. Investigating hospital administrators’ experience of workplace mistreatment. Can J Behav Sci. 2005;37:262-72.
187. Harris EG, Artis AB, Walters JH, Licata JW. Role stressors, service worker job resourcefulness, and job outcomes: An empirical analysis. J Bus Res. 2006;59:407-15.
188. Harris KJ, Andrews MC, Kacmar KM. The moderating effects of justice on the relationship between organizational politics and workplace attitudes. J Bus Psychol. 2007;22:135-44.
189. Harris KJ, Harvey P, Kacmar KM. Do social stressors impact everyone equally? An examination of the moderating impact of core self-evaluations. J Bus Psychol. 2009;24:153-64.
190. Harris KJ, Harvey P, Kacmar KM. Abusive supervisory reactions to coworker relationship conflict. Leadersh Q. 2011;22:1010-23.
191. Harris RB, Harris KJ, Harvey P. A test of competing models of the relationships among perceptions of organizational politics, perceived organizational support, and individual outcomes. J Soc Psychol. 2007;147:631-56.
192. Harvey P, Harris RB, Harris KJ, Wheeler AR. Attenuating the effects of social stress: The impact of political skill. J Occup Health Psychol. 2007;12:105-15.
193. Harvey P, Stoner J, Hochwarter W, Kacmar C. Coping with abusive supervision: The neutralizing effects of ingratiation and positive affect on negative employee outcomes. Leadersh Q. 2007;18:264-80.
194. Harvey S, Blouin C, Stout D. Proactive personality as a moderator of outcomes for young workers experiencing conflict at work. Pers Individ Differ. 2006;40:1063-74.
195. Hassan S, Wright BE, Yukl G. Does ethical leadership matter in government? Effects on organizational commitment, absenteeism, and willingness to report ethical problems. Public Adm Rev. 2014;74:333-43.
196. Hauge LJ, Skogstad A, Einarsen S. Relationships between stressful work environments and bullying: Results of a large representative study. Work Stress. 2007;21:220-42.
197. Hauge LJ, Skogstad A, Einarsen S. The relative impact of workplace bullying as a social stressor at work. Scand J Psychol. 2010;51:426-33.
198. Hausknecht JP, Sturman MC, Roberson QM. Justice as a dynamic construct: Effects of individual trajectories on distal work outcomes. J Appl Psychol. 2011;96:872-80.
199. He H, Zhu W, Zheng X. Procedural justice and employee engagement: Roles of organizational identification and moral identity centrality. J Bus Ethics. 2014;122:681-95.
200. Heinisch DA, Jex SM. Negative affectivity and gender as moderators of the relationships between work-related stressors and depressed mood at work. Work Stress. 1997;11:46-57.
201. Hendrix WH, Robbins T, Miller J, Summers TP. Effects of procedural and distributive justice on factors predictive of turnover. J Soc Behav Pers. 1998;13:611-32.
202. Hershcovis MS, Reich TC, Parker SK, Bozeman J. The relationship between workplace aggression and target deviant behaviour: The moderating roles of power and task interdependence. Work Stress. 2012;26:1-20.
203. Hesson-McInnis MS, Fitzgerald LF. Sexual harassment: A preliminary test of an integrative model. J Appl Soc Psychol. 1997;27:877-901.
204. Ho VT. Co‐worker mistreatment in a Singaporean Chinese firm: The roles of third party embeddedness and network closure. Manag Organ Rev. 2014;10:109-34.
205. Hogh A, Borg V, Mikkelsen KL. Work-related violence as a predictor of fatigue: A 5-year follow-up of the Danish work environment cohort study. Work Stress. 2003;17:182-94.
206. Hogh A, Henriksson ME, Burr H. A 5-year follow-up study of aggression at work and psychological health. Int J Behav Med. 2005;12:256-65.
207. Hogh A, Hoel H, Carneiro IG. Bullying and employee turnover among healthcare workers: A three‐wave prospective study. J Nurs Manag. 2011;19:742-51.
208. Hogh A, Mikkelsen EG. Is sense of coherence a mediator or moderator of relationships between violence at work and stress reactions? Scand J Psychol. 2005;46:429-37.
209. Holmvall CM, Sidhu J. Predicting customer service employees’ job satisfaction and turnover intentions: The roles of customer interactional injustice and interdependent self-construal. Soc Justice Res. 2007;20:479-96.
210. Holtz BC, Harold CM. Effects of leadership consideration and structure on employee perceptions of justice and counterproductive work behavior. J Organ Behav. 2013;34: 492-519.
211. Holtz BC, Harold CM. Interpersonal justice and deviance: The moderating effects of interpersonal justice values and justice orientation. J Manag. 2013;39:339-65.
212. Hon AHY, Lu L. The mediating role of trust between expatriate procedural justice and employee outcomes in Chinese hotel industry. Int J Hosp Manag. 2010;29:669-76.
213. Hoobler JM, Brass DJ. Abusive supervision and family undermining as displaced aggression. J Appl Psychol. 2006;91:1125-33.
214. Hoobler JM, Hu J. A model of injustice, abusive supervision, and negative affect. Leadersh Q. 2013;24:256-69.
215. Hoobler JM, Rospenda KM, Lemmon G, Rosa JA. A within-subject longitudinal study of the effects of positive job experiences and generalized workplace harassment on well-being. J Occup Health Psychol. 2010;15:434-51.
216. Hoppe A. Psychosocial working conditions and well-being among immigrant and German low-wage workers. J Occup Health Psychol. 2011a;16:187-201.
217. Hoppe A. Stressbezogene Arbeitsanalyse bei kultureller Diversität: Entwicklung eines Screeninginstruments für interkulturelle Belegschaften in un- und angelernten Berufen [Stress-related job analysis for culturally diverse workplaces: Developing a survey instrument for a culturally diverse and low-skilled workforce]. Z Arb Organ. 2011b;55:17-31.
218. Howard LW, Cordes CL. Flight from unfairness: Effects of perceived injustice on emotional exhaustion and employee withdrawal. J Bus Psychol. 2010;25:409-28.
219. Huang I-C, Chuang C-HJ, Lin H-C. The role of burnout in the relationship between perceptions of organizational politics and turnover intentions. Public Pers Manag. 2003;32:519-31.
220. Huebner ES. Burnout among school psychologists: An exploratory investigation into its nature, extent, and correlates. Sch Psychol Q. 1992;7:129-36.
221. Hunter EM, Penney LM. The waiter spit in my soup! Antecedents of customer-directed counterproductive work behavior. Hum Perform. 2014;27:262-81.
222. Ilie A, Penney LM, Ispas D, Iliescu D. The role of trait anger in the relationship between stressors and cunterproductive work behaviors: Convergent findings from multiple studies and methodologies. Appl Psychol-Int Rev. 2012;61:415-36.
223. Induresan J. Some correlates of perception of organizational climate. Manag Psychol. 1981;2:40-43.
224. Jacobs G, Belschak FD, Den Hartog DN. (Un)ethical behavior and performance appraisal: The role of affect, support, and organizational justice. J Bus Ethics. 2014;121:63-76.
225. Jacobsson C, Pousette A, Thylefors I. Managing stress and feelings of mastery among Swedish comprehensive school teachers. Scand J Educ Res, 2001;45:37-53.
226. Jafari P, Bidarian S. The relationship between organizational justice and organizational citizenship behavior. Procedia Soc Behav Sci. 2012;47:1815-20.
227. Jain AK, Cooper CL. Stress and organisational citizenship behaviours in Indian business process outsourcing organisations. IIMB Manag Rev. 2012;24:155-63.
228. Jakopec A, Sušanj Z. Effects of (Mis)alignment between supervisory and organizational justice. Druš Istraž-J Gen Soc Issues. 2014;23:615-37.
229. Janssen O. Innovative behaviour and job involvement at the price of conflict and less satisfactory relations with co-workers. J Occup Organ Psychol. 2003;76:347-64.
230. Janssen O, Lam CK, Huang X. Emotional exhaustion and job performance: The moderating roles of distributive justice and positive affect. J Organ Behav. 2010;31:787-809.
231. Jaramillo F, Mulki JP, Boles JS. Workplace stressors, job attitude, and job behaviors: Is interpersonal conflict the missing link? J Pers Sell Sales Manag. 2011;31:339-56.
232. Jehn KA. A multimethod examination of the benefits and detriments of intragroup conflict. Adm Sci Q. 1995;40:256-82.
233. Jehn KA, Northcraft GB, Neale MA. Why differences make a difference: A field study of diversity, conflict, and performance in workgroups. Adm Sci Q. 1999;44:741-63.
234. Jepsen DM, Rodwell J. Female perceptions of organizational justice. Gend Work Organ. 2012;19:723-40.
235. Jex SM, Beehr TA, Roberts CK. The meaning of occupational stress items to survey respondents. J Appl Psychol. 1992;77:623-28.
236. Jex SM, Spector PE. The impact of negative affectivity on stressor-strain relations: A replication and extension. Work Stress. 1996;10:36-45.
237. Jian G. Revisiting the association of LMX quality with perceived role stressors: Evidence for inverted U relationships among immigrant employees. Commun Res. 2014;41:52-73.
238. Jiang D-Y, Cheng B-S. Affect‐ and role‐based loyalty to supervisors in Chinese organizations. Asian J Soc Psychol. 2008;11:214-21.
239. Jiménez BM, Muñoz AR, Gamarra MM, Herrer MG. Assessing workplace bullying: Spanish validation of a reduced version of the Negative Acts Questionnaire. Span J Psychol. 2007;10:449-57.
240. Jockin V, Arvey RD, McGue M. Perceived victimization moderates self-reports of workplace aggression and conflict. J Appl Psychol. 2001;86:1262-9.
241. Johnson JS, Sohi RS. The curvilinear and conditional effects of product line breadth on salesperson performance, role stress, and job satisfaction. J Acad Mark Sci. 2014;42:71-89.
242. Johnson RE, Selenta C, Lord RG. When organizational justice and the self-concept meet: Consequences for the organization and its members. Organ Behav Hum Decis Process. 2006;99:175-201.
243. Jones DA. Getting even with one’s supervisor and one’s organization: Relationships among types of injustice, desires for revenge, and counterproductive work behaviors. J Organ Behav. 2009;30:525-42.
244. Judge TA, Colquitt JA. Organizational justice and stress: The mediating role of work–family conflict. J Appl Psychol. 2004;89:395-404.
245. Judge TA, Scott BA, Ilies R. Hostility, job attitudes, and workplace deviance: Test of a multilevel model. J Appl Psychol. 2006;91:126-38.
246. Jung HS, Yoon HH, Kim YJ. Effects of culinary employees’ role stress on burnout and turnover intention in hotel industry: Moderating effects on employees’ tenure. Serv Ind J. 2012;32:2145-65.
247. Kälin W, Semmer NK, Elfering A, Tschan F, Dauwalder J-P, Heunert S, Crettaz von Roten F. Work characteristics and well-being of Swiss apprentices entering the labor market. Swiss J Psychol. 2000;59:272-90.
248. Kamdar D, McAllister DJ, Turban DB. “All in a day’s work”: How follower individual differences and justice perceptions predict OCB role definitions and behavior. J Appl Psychol. 2006;91:841-55.
249. Karatepe OM, Nkendong RA. The relationship between customer-related social stressors and job outcomes: The mediating role of emotional exhaustion. Econ Res-Ekon Istraž. 2014;27:414-26.
250. Karatepe OM, Shahriari S. Job embeddedness as a moderator of the impact of organisational justice on turnover intentions: A study in Iran. Int J Tour Res., 2014;16:22-32.
251. Karatepe OM, Sokmen A. The effects of work role and family role variables on psychological and behavioral outcomes of frontline employees. Tour Manag. 2006;27:255-68.
252. Karatepe OM, Uludag O. Role stress, burnout and their effects on frontline hotel employees’ job performance: Evidence from Northern Cyprus. Int J Tour Res. 2008;10:111-26.
253. Karriker J. Justice as strategy: The role of procedural justice in an organizational realignment. J Chang Manag. 2007;7: 329-42.
254. Karriker JH, Williams ML Organizational justice and organizational citizenship behavior: A mediated multifoci model. J Manag. 2009;35:112-35.
255. Kath LM, Swody CA, Magley VJ, Bunk JA, Gallus JA. Cross-level, three-way interactions among work-group climate, gender, and frequency of harassment on morale and withdrawal outcomes of sexual harassment. J Occup Organ Psychol. 2009;82:159-82.
256. Kaukiainen A, Salmivalli C, Björkqvist K, Österman K, Lahtinen A, Kostamo A, Lagerspetz K. Overt and covert aggression in work settings in relation to the subjective well-being of employees. Aggress Behav. 2001;27:360-71.
257. Kausto J, Elo A-L, Lipponen J, Elovainio M. Moderating effects of job insecurity in the relationships between procedural justice and employee well-being: Gender differences. Eur J Work Organ Psychol. 2005;14:431-52.
258. Keashly L, Hunter S, Harvey S. Abusive interaction and role state stressors: Relative impact on student residence assistant stress and work attitudes. Work Stress. 1997;11:175-85.
259. Keashly L, Trott V, MacLean LM. Abusive behavior in the workplace: A preliminary investigation. Violence Vict. 1994;9:341-57.
260. Kern JH, Grandey AA. Customer incivility as a social stressor: The role of race and racial identity for service employees. J Occup Health Psychol. 2009;14:46-57.
261. Kernan MC, Hanges PJ. Survivor reactions to reorganization: Antecedents and consequences of procedural, interpersonal, and informational justice. J Appl Psychol. 2002;87:916-28.
262. Kessler SR, Bruursema K, Rodopman B, Spector PE. Leadership, interpersonal conflict, and counterproductive work behavior: An examination of the stressor–strain process. Negot Confl Manag Res. 2013;6:180-90.
263. Kessler SR, Spector PE, Chang C-H, Parr AD. Organizational violence and aggression: Development of the three-factor Violence Climate Survey. Work Stress. 2008;22:108-24.
264. Khan AK, Quratulain S, Crawshaw JR. The mediating role of discrete emotions in the relationship between injustice and counterproductive work behaviors: A study in Pakistan. J Bus Psychol. 2013;28:49-61.
265. Kickul J, Lester SW, Finkl J. Promise breaking during radical organizational change: Do justice interventions make a difference? J Organ Behav. 2002;23:469-88.
266. Kidwell Jr RE, Bennett N. Employee reactions to electronic control systems: The role of procedural fairness. Group Organ Manag. 1994;19:203-18.
267. Kim H-S. Examining the role of informational justice in the wake of downsizing from an organizational relationship management perspective. J Bus Ethics. 2009;88:297-312.
268. Kim T-Y, Leung K. Forming and reacting to overall fairness: A cross-cultural comparison. Organ Behav Hum Decis Process. 2007;104:83-95.
269. King RC, Xia W, Campbell Quick J, Sethi V. Socialization and organizational outcomes of information technology professionals. Career Dev Int. 2005;10:26-51.
270. Kinman G. Work stressors, health and sense of coherence in UK academic employees. Educ Psychol. 2008;28:823-35.
271. Kisamore JL, Jawahar IM, Liguori EW, Mharapara TL, Stone TH. Conflict and abusive workplace behaviors: The moderating effects of social competencies. Career Dev Int. 2010;15:583-600.
272. Kissman K. Women in blue-collar occupations: An exploration of constraints and facilitators. J Sociol Soc Welf. 1990;17:139-49.
273. Kitaoka-Higashiguchi K. Burnout as a developmental process among Japanese nurses: Investigation of Leiter’s model. Jpn J Nurs Sci. 2005;2:9-16.
274. Kivimäki M, Lindström K. Effects of private self‐consciousness and control on the occupational stress‐strain relationship. Stress Med. 1995;11:7-16.
275. Klainin P. Stress and health outcomes: The mediating role of negative affectivity in female health care workers. Int J Stress Manag. 2009;16:45-64.
276. Kokkinos CM. Job stressors, personality and burnout in primary school teachers. Br J Educ Psychol. 2007;77:229-43.
277. Korsgaard MA, Sapienza HJ, Schweiger DM. Beaten before begun: The role of procedural justice in planning change. J Manag. 2002;28:497-516.
278. Krischer MM, Penney LM, Hunter EM. Can counterproductive work behaviors be productive? CWB as emotion-focused coping. J Occup Health Psychol. 2010;15:154-66.
279. Kuvaas B. An exploration of how the employee–organization relationship affects the linkage between perception of developmental human resource practices and employee outcomes. J Manag Stud. 2008;45:1-25.
280. Kwon S, Kim MS, Kang S-C, Kim MU. Employee reactions to gainsharing under seniority pay systems: The mediating effect of distributive, procedural, and interactional justice. Hum Resour Manag. 2008;47:757-75.
281. Kwong JYY, Leung K. A moderator of the interaction effect of procedural justice and outcome favorability: Importance of the relationship. Organ Behav Hum Decis Process. 2002;87:278-99.
282. Ladebo OJ, Awotunde JM, AbdulSalaam-Saghir P. Coworkers’ and supervisor interactional justice: Correlates of extension personnel’s job satisfaction, distress, and aggressive behavior. J Behav Appl Manag. 2008;9:206-25.
283. Lam LW, Loi R, Leong C. Reliance and disclosure: How supervisory justice affects trust in supervisor and extra-role performance. Asia Pac J Manag. 2013;30:231-49.
284. Lam SSK, Schaubroeck J, Aryee S. Relationship between organizational justice and employee work outcomes: A cross‐national study. J Organ Behav. 2002;23:1-18.
285. Lambert EG, Hogan NL, Cheeseman K, Barton‐Bellessa SM. The relationship between job stressors and job involvement among correctional staff: A test of the job strain model. Howard J Crim Justice. 2013;52:19-38.
286. Lankau MJ, Carlson DS, Nielson TR. The mediating influence of role stressors in the relationship between mentoring and job attitudes. J Vocat Behav. 2006;68:308-22.
287. Laschinger HK, Wong C, Regan S, Young-Ritchie C, Bushell P. Workplace incivility and new graduate nurses’ mental health: The protective role of resiliency. J Nurs Adm. 2013;43:415-21.
288. Laschinger HKS, Grau AL, Finegan J, Wilk P. New graduate nurses’ experiences of bullying and burnout in hospital settings. J Adv Nurs. 2010;66:2732-42.
289. Laschinger HKS, Leiter M, Day A, Gilin D. Workplace empowerment, incivility, and burnout: Impact on staff nurse recruitment and retention outcomes. J Nurs Manag. 2009;17:302-11.
290. Lavelle JJ, Brockner J, Konovsky MA, Price KH, Henley AB, Taneja A, Vinekar V. Commitment, procedural fairness, and organizational citizenship behavior: A multifoci analysis. J Organ Behav. 2009;30:337-57.
291. Law R, Dollard MF, Tuckey MR, Dormann C. Psychosocial safety climate as a lead indicator of workplace bullying and harassment, job resources, psychological health and employee engagement. Accid Anal Prev. 2011;43:1782-93.
292. Lawrence ER, Kacmar KM. Leader-member exchange and stress: The mediating role of job involvement and role conflict. J Behav Appl Manag. 2012;14:39-52.
293. Le Roy J, Bastounis M, Minibas-Poussard J. Interactional justice and counterproductive work behaviors: The mediating role of negative emotions. Soc Behav Pers-Int J. 2012;40:1341-55.
294. LeBlanc MM, Kelloway EK. Predictors and outcomes of workplace violence and aggression. J Appl Psychol. 2002;87:444-53.
295. Lee C, Farh J-L. The effects of gender in organizational justice perception. J Organ Behav. 1999;20:133-43.
296. Lee JSY, Akhtar S. Effects of the workplace social context and job content on nurse burnout. Hum Resour Manag. 2011;50:227-45.
297. Lee RT, Brotheridge CM. When prey turns predatory: Workplace bullying as a predictor of counteraggression/bullying, coping, and well-being. Eur J Work Organ Psychol. 2006;15:352-77.
298. Leiter MP, Frizzell C, Harvie P, Churchill L. Abusive interactions and burnout: Examining occupation, gender, and the mediating role of community. Psychol Health. 2001;16:547-63.
299. Leiter MP, Price SL, Laschinger HKS. Generational differences in distress, attitudes and incivility among nurses. J Nurs Manag. 2010;18:970-80.
300. Leung K, Wang Z, Smith PB. Job attitudes and organizational justice in joint venture hotels in China: The role of expatriate managers. Int J Hum Resour Manag. 2001;12:926-45.
301. Li F, Yu KF, Yang J, Qi Z, Fu JH-Y. Authentic leadership, traditionality, and interactional justice in the Chinese context. Manag Organ Rev. 2014;10:249-73.
302. Li F, Zhou F, Leung K. Expecting the worst: Moderating effects of social cynicism on the relationships between relationship conflict and negative affective reactions. J Bus Psychol. 2011;26:339-45.
303. Li H, Bingham JB, Umphress EE. Fairness from the top: Perceived procedural justice and collaborative problem solving in new product development. Organ Sci. 2007;18:200-16.
304. Li Y. Building affective commitment to organization among Chinese university teachers: The roles of organizational justice and job burnout. Educ Assess Eval Acc. 2014;26:135-52.
305. Liao H, Rupp DE. The impact of justice climate and justice orientation on work outcomes: A cross-level multifoci framework. J Appl Psychol. 2005;90:242-56.
306. Liljegren M, Ekberg K. The longitudinal relationship between job mobility, perceived organizational justice, and health. BMC Public Health. 2008;8:164.
307. Lim S, Cortina LM. Interpersonal mistreatment in the workplace: The interface and impact of general incivility and sexual harassment. J Appl Psychol. 2005;90:483-96.
308. Lim S, Cortina LM, Magley VJ. Personal and workgroup incivility: Impact on work and health outcomes. J Appl Psychol. 2008;93:95-107.
309. Lim S, Lee A. Work and nonwork outcomes of workplace incivility: Does family support help? J Occup Health Psychol. 2011;16:95-111.
310. Lim VKG. The IT way of loafing on the job: Cyberloafing, neutralizing and organizational justice. J Organ Behav. 2002;23:675-94.
311. Lim VKG, Teo TSH. Mind your e-manners: Impact of cyber incivility on employees’ work attitude and behavior. Inf Manag. 2009;46:419-25.
312. Lim VKG, Yuen EC. Doctors, patients, and perceived job image: An empirical study of stress and nurses in Singapore. J Behav Med. 1998;21:269-82.
313. Lin C-P, Hung W-T, Chiu C-K. Being good citizens: Understanding a mediating mechanism of organizational commitment and social network ties in OCBs. J Bus Ethics. 2008;81:561-78.
314. Lindblom KM, Linton SJ, Fedeli C, Bryngelsson I-L. Burnout in the working population: Relations to psychosocial work factors. Int J Behav Med. 2006;13:51-9.
315. Liu C, Nauta MM, Spector PE, Li C. Direct and indirect conflicts at work in China and the US: A cross-cultural comparison. Work Stress. 2008;22:295-313.
316. Liu C, Spector PE, Shi L. Cross-national job stress: A quantitative and qualitative study. J Organ Behav. 2007;28:209-39.
317. Liu C, Spector PE, Shi L. Use of both qualitative and quantitative approaches to study job stress in different gender and occupational groups. J Occup Health Psychol. 2008;13:357-70.
318. Liu D, Liao H, Loi R. The dark side of leadership: A three-level investigation of the cascading effect of abusive supervision on employee creativity. Acad Manag J. 2012;55:1187-212.
319. Liu JY-C, Chen H-G, Chen CC, Sheu TS. Relationships among interpersonal conflict, requirements uncertainty, and software project performance. Int J Proj Manag. 2011;29:547-56.
320. Liu X-Y, Kwan HK, Chiu RK. Customer sexual harassment and frontline employees’ service performance in China. Hum Relat. 2014;67:333-56.
321. Liu X-Y, Wang J. Abusive supervision and organizational citizenship behaviour: Is supervisor–subordinate guanxi a mediator? Int J Hum Resour Manag. 2013;24:1471-89.
322. Loh J, Restubog SLD, Zagenczyk TJ. Consequences of workplace bullying on employee identification and satisfaction among Australians and Singaporeans. J Cross Cult Psychol. 2010;41:236-52.
323. Loi R, Hang‐yue N, Foley S. Linking employees’ justice perceptions to organizational commitment and intention to leave: The mediating role of perceived organizational support. J Occup Organ Psychol. 2006;79:101-20.
324. Lowe RH, Vodanovich SJ. A field study of distributive and procedural justice as predictors of satisfaction and organizational commitment. J Bus Psychol. 1995;10:99-114.
325. Lu C-Q, Siu O-L, Au W-T, Leung SSW. Manager’s occupational stress in state-owned and private enterprises in the People’s Republic of China. Int J Hum Resour Manag. 2009;20:1670-82.
326. Lu L, Chang Y-Y, Lai SY-L. What differentiates success from strain: The moderating effects of self-efficacy. Int J Stress Manag. 2011;18:396-412.
327. Lu L, Kao S-F, Siu O-L, Lu C-Q. Work stress, Chinese work values, and work well-being in the Greater China. J Soc Psychol. 2011;151:767-83.
328. Lu L, Siu O-L, Lu C-Q. Does loyalty protect Chinese workers from stress? The role of affective organizational commitment in the Greater China Region. Stress Health. 2010;26:161-8.
329. Luo Z, Song H, Marnburg E, Øgaard T. The impact of relational identity on the relationship between LMX, interpersonal justice, and employees’ group commitment. Int J Hosp Manag. 2014;41:21-7.
330. Lutgen-Sandvik P, Tracy SJ, Alberts JK. Burned by bullying in the American workplace: Prevalence, perception, degree and impact. J Manag Stud. 2007;44:837-62.
331. Ma B, Liu S, Liu D. The impact of organizational identification on the relationship between procedural justice and employee work outcomes. Soc Behav Pers. 2014;42:437-44.
332. Mackey JD, Ellen III BP, Hochwarter WA, Ferris GR. Subordinate social adaptability and the consequences of abusive supervision perceptions in two samples. Leadersh Q. 2013;24:732-46.
333. Magley VJ, Hulin CL, Fitzgerald LF, DeNardo M. Outcomes of self-labeling sexual harassment. J Appl Psychol. 1999;84:390-402.
334. Maier GW, Streicher B, Jonas E, Woschée R. Gerechtigkeitseinschätzungen in Organisationen: Die Validität einer deutschsprachigen Fassung des Fragebogens von Colquitt (2001) [Assessment of justice in organizations: The validity of a German version of the questionnaire by Colquitt (2001)]. Diagnostica. 2007;53:97-108.
335. Marcus B, Schuler H. Antecedents of counterproductive behavior at work: A general perspective. J Appl Psychol. 2004;89:647-60.
336. Martin AJ, Jones ES, Callan VJ. The role of psychological climate in facilitating employee adjustment during organizational change. Eur J Work Organ Psychol. 2005;14:263-89.
337. Martin RJ, Hine DW. Development and validation of the Uncivil Workplace Behavior Questionnaire. J Occup Health Psychol. 2005;10:477-90.
338. Martinussen M, Richardsen AM. Air traffic controller burnout: Survey responses regarding job demands, job resources, and health. Aviat Space Environ Med. 2006;77:422-28.
339. Masterson SS. A trickle-down model of organizational justice: Relating employees’ and customers’ perceptions of and reactions to fairness. J Appl Psychol. 2001;86:594-604.
340. Masterson SS, Lewis K, Goldman BM, Taylor MS. Integrating justice and social exchange: The differing effects of fair procedures and treatment on work relationships. Acad Manag J. 2000;43:738-48.
341. Mathisen GE, Einarsen S, Mykletun R. The occurrences and correlates of bullying and harassment in the restaurant sector. Scand J Psychol. 2008;49:59-68.
342. Matta FK, Erol‐Korkmaz HT, Johnson RE, Biçaksiz P. Significant work events and counterproductive work behavior: The role of fairness, emotions, and emotion regulation. J Organ Behav. 2014;35:920-44.
343. Mayer D, Nishii L, Schneider B, Goldstein H. The precursors and products of justice climates: Group leader antecedents and employee attitudinal consequences. Pers Psychol. 2007;60:929-63.
344. McCormack D, Casimir G, Djurkovic N, Yang L. The concurrent effects of workplace bullying, satisfaction with supervisor, and satisfaction with co-workers on affective commitment among schoolteachers in China. Int J Confl Manag. 2006;17:316-31.
345. McGonagle AK, Barnes‐Farrell JL. Chronic illness in the workplace: Stigma, identity threat and strain. Stress Health. 2014;30:310-21.
346. McLaney MA, Hurrell Jr JJ. Control, stress, and job satisfaction in Canadian nurses. Work Stress. 1988;2:217-24.
347. McNall LA, Roch SG. A social exchange model of employee reactions to electronic performance monitoring. Hum Perform. 2009;22:204-24.
348. Medler-Liraz H, Kark R. It takes three to tango: Leadership and hostility in the service encounter. Leadersh Q. 2012;23:81-93.
349. Meier LL, Gross S, Spector PE, Semmer NK. Relationship and task conflict at work: Interactive short-term effects on angry mood and somatic complaints. J Occup Health Psychol. 2013;18:144-56.
350. Meier LL, Spector PE. Reciprocal effects of work stressors and counterproductive work behavior: A five-wave longitudinal study. J Appl Psychol. 2013;98:529-39.
351. Meliá JL, Becerril M. Psychosocial sources of stress and burnout in the construction sector: A structural equation model. Psicothema, 2007;19:679-86.
352. Menon S, Narayanan L, Spector PE. Time urgency and its relation to occupational stressors and health outcomes for health care professionals. In: Spielberger CD, Sarason IG, editors. Stress and Emotion: Anxiety, Anger, and Curiosity. Vol 16. Washington: Taylor & Francis; 1996. p. 127-42.
353. Meurs JA, Fox S, Kessler SR, Spector PE. It’s all about me: The role of narcissism in exacerbating the relationship between stressors and counterproductive work behaviour. Work Stress. 2013;27:368-82.
354. Michel A, Stegmaier R, Sonntag K. I scratch your back – you scratch mine. Do procedural justice and organizational identification matter for employees’ cooperation during change? J Chang Manag. 2010;10:41-59.
355. Mikkelsen EG, Einarsen S. Bullying in Danish work-life: Prevalence and health correlates. Eur J Work Organ Psychol. 2001;10:393-413.
356. Mikkelsen EG, Einarsen S. Relationships between exposure to bullying at work and psychological and psychosomatic health complaints: The role of state negative affectivity and generalized self-efficacy. Scand J Psychol. 2002;43:397-405.
357. Miles DE, Borman WE, Spector PE, Fox S. Building an integrative model of extra role work behaviors: A comparison of counterproductive work behavior with organizational citizenship behavior. Int J Sel Assess. 2002;10:51-7.
358. Miner KN, Pesonen AD, Smittick AL, Seigel ML, Clark EK. Does being a mom help or hurt? Workplace incivility as a function of motherhood status. J Occup Health Psychol. 2014;19:60-73.
359. Miner KN, Settles IH, Pratt-Hyatt JS, Brady CC. Experiencing incivility in organizations: The buffering effects of emotional and organizational support. J Appl Soc Psychol. 2012;42:340-72.
360. Miner-Rubino K, Cortina LM. Working in a context of hostility toward women: Implications for employees’ well-being. J Occup Health Psychol. 2004;9:107-22.
361. Miner-Rubino K, Reed WD. Testing a moderated mediational model of workgroup incivility: The roles of organizational trust and group regard. J Appl Soc Psychol. 2010;40:3148-68.
362. Mingzheng W, Xiaoling S, Xubo F, Youshan L. Moral identity as a moderator of the effects of organizational injustice on counterproductive work behavior among Chinese public servants. Public Pers Manag. 2014;43:314-24.
363. Mitchell MS, Ambrose ML. Abusive supervision and workplace deviance and the moderating effects of negative reciprocity beliefs. J Appl Psychol. 2007;92:1159-68.
364. Mohamed I, Yeo RK. Impact of organisational justice on work outcomes in the pharmaceutical industry in Kuwait. Int J Hum Resour Dev Manag. 2014;14:17-46.
365. Moideenkutty U, Blau G, Kumar R, Nalakath A. Comparing correlates of organizational citizenship versus in-role behavior of sales representatives in India. Int J Comm Manag. 2006;16:15-28.
366. Moliner C, Martínez-Tur V, Peiró JM, Ramos J. Linking organizational justice to burnout: Are men and women different? Psychol Rep. 2005;96:805-16.
367. Moliner C, Martínez‐Tur V, Peiró JM, Ramos J, Cropanzano R. Perceived reciprocity and well‐being at work in non‐professional employees: Fairness or self‐interest? Stress Health. 2013;29:31-9.
368. Moorman RH. Relationship between organizational justice and organizational citizenship behaviors: Do fairness perceptions influence employee citizenship? J Appl Psychol. 1991;76:845-55.
369. Moorman RH, Blakely GL, Niehoff BP. Does perceived organizational support mediate the relationship between procedural justice and organizational citizenship behavior? Acad Manag J. 1998;41:351-7.
370. Moreno-Jiménez B, Rodríguez-Muñoz A, Pastor JC, Sanz-Vergel AI, Garrosa E. The moderating effects of psychological detachment and thoughts of revenge in workplace bullying. Pers Individ Differ. 2009;46:359-64.
371. Morrow PC, McElroy JC, Phillips CM. Sexual harassment behaviors and work related perceptions and attitudes. J Vocat Behav. 1994;45:295-309.
372. Mossholder KW, Bennett N, Kemery ER, Wesolowski MA. Relationships between bases of power and work reactions: The mediational role of procedural justice. J Manag. 1998;24:533-52.
373. Mueller S, Tschan, F. Consequences of client-initiated workplace violence: The role of fear and perceived prevention. J Occup Health Psychol. 2011;16:217-29.
374. Mulki JP, Locander WB, Marshall GW, Harris EG, Hensel J. Workplace isolation, salesperson commitment, and job performance. J Pers Sell Sales Manag. 2008;28:67-78.
375. Murphy C, Ramamoorthy N, Flood PC, MacCurtain S. Organizational justice perceptions and employee attitudes among Irish blue collar employees: An empirical test of the main and moderating roles of individualism/collectivism. Manag Rev. 2006;17:328-43.
376. Murray-Gibbons R, Gibbons C. Occupational stress in the chef profession. Int J Contemp Hosp Manag. 2007;19:32-42.
377. Nadiri H, Tanova C. An investigation of the role of justice in turnover intentions, job satisfaction, and organizational citizenship behavior in hospitality industry. Int J Hosp Manag. 2010;29:33-41.
378. Nakata A, Haratani T, Takahashi M, Kawakami N, Arito H, Kobayashi F, Araki S. Job stress, social support, and prevalence of insomnia in a population of Japanese daytime workers. Soc Sci Med. 2004;59:1719-30.
379. Nandkeolyar AK, Shaffer JA, Li A, Ekkirala S, Bagger J. Surviving an abusive supervisor: The joint roles of conscientiousness and coping strategies. J Appl Psychol. 2014;99:138-50.
380. Nasurdin AM, Khuan SL. Organizational justice as an antecedent of job performance. Gadjah Mada Int J Bus. 2007;9:335-53.
381. Newton CJ, Jimmieson NL. Subjective fit with organizational culture: An investigation of moderating effects in the work stressor-employee adjustment relationship. Int J Hum Resour Manag. 2009;20:1770-89.
382. Ngo HY, Loi R, Foley S, Zheng X, Zhang L. Perceptions of organizational context and job attitudes: The mediating effect of organizational identification. Asia Pac J Manag. 2013;30:149-68.
383. Niehoff BP, Moorman RH. Justice as a mediator of the relationship between methods of monitoring and organizational citizenship behavior. Acad Manag J. 1993;36:527-56.
384. Nielsen MB, Bergheim K, Eid J. Relationships between work environment factors and workers’ well-being in the maritime industry. Int Marit Health. 2013;64:80-8.
385. Nijman H, Bowers L, Oud N, Jansen G. Psychiatric nurses’ experiences with inpatient aggression. Aggress Behav. 2005;31:217-27.
386. Noblet A, Maharee-Lawler S, Rodwell J. Using job strain and organizational justice models to predict multiple forms of employee performance behaviours among Australian policing personnel. Int J Hum Resour Manag. 2012;23:3009-26.
387. O’Connell CE, Korabik K. Sexual harassment: The relationship of personal vulnerability, work context, perpetrator status, and type of harassment to outcomes. J Vocat Behav. 2000;56:299-329.
388. Olkkonen M-E, Lipponen J. Relationships between organizational justice, identification with organization and work unit, and group-related outcomes. Organ Behav Hum Decis Process. 2006;100:202-15.
389. Oluwafemi OJ. Predictors of turnover intention among employees in Nigeria’s oil industry. Organ Mark Emerg Econ. 2013;4:42-63.
390. O’Neill TA, Lewis RJ, Carswell JJ. Employee personality, justice perceptions, and the prediction of workplace deviance. Pers Individ Differ. 2011;51:595-600.
391. Oren L, Tziner A, Nahshon Y, Sharoni G. Relations between OCBs, organizational justice, work motivation and self-efficacy. Amfiteatru Econ. 2013;15:505-17.
392. Orthmann A, Gunkel L, Otte R. Ressourcen als Schlüssel für Führung und Gesundheit im Betrieb [Resources as a key for leadership and health at work]. In: Badura B, Ducki A, Schröder H, Klose J, Macco K, editors. Fehlzeiten-Report 2011: Führung und Gesundheit: Zahlen Daten, Analysen aus allen Branchen der Wirtschaft. Berlin: Springer; 2011, p. 135-46.
393. Otto K, Schmidt S. Dealing with stress in the workplace: Compensatory effects of belief in a just world. Eur Psychol. 2007;12:272-82.
394. Pangert B, Schüpbach H. Arbeitsbedingungen und Gesundheit von Führungskräften auf mittlerer und unterer Hierarchieebene [Working conditions and health of leaders at middle and lower hierarchical levels]. In: Badura B, Ducki A, Schröder H, Klose J, Macco K, editors. Fehlzeiten-Report 2011: Führung und Gesundheit: Zahlen Daten, Analysen aus allen Branchen der Wirtschaft. Berlin: Springer; 2011, p, 71-9.
395. Paré G, Tremblay M. The influence of high-involvement human resources practices, procedural justice, organizational commitment, and citizenship behaviors on information technology professionals’ turnover intentions. Group Organ Manag. 2007;32:326-57.
396. Parker RJ, Kohlmeyer III JM. Organizational justice and turnover in public accounting firms: A research note. Account Organ Soc. 2005;30:357-69.
397. Patel C, Budhwar P, Varma A. Overall justice, work group identification and work outcomes: Test of moderated mediation process. J World Bus. 2012;47:213-22.
398. Peng J-C, Chiu S-F. An integrative model linking feedback environment and organizational citizenship behavior. J Soc Psychol. 2010;150:582-607.
399. Peng J-C, Tseng M-M, Lee Y-L. Relationships among supervisor feedback environment, work-related stressors, and employee deviance. J Nurs Res. 2011;19:13-24.
400. Penhaligon NL, Louis WR, Restubog SLD. Emotional anguish at work: The mediating role of perceived rejection on workgroup mistreatment and affective outcomes. J Occup Health Psychol. 2009;14:34-45.
401. Penney LM, Spector PE. Job stress, incivility, and counterproductive work behavior (CWB): The moderating role of negative affectivity. J Organ Behav. 2005;26:777-96.
402. Piccolo RF, Bardes M, Mayer DM, Judge TA. Does high quality leader–member exchange accentuate the effects of organizational justice? Eur J Work Organ Psychol. 2008;17:273-98.
403. Pillai R, Schriesheim CA, Williams ES. Fairness perceptions and trust as mediators for transformational and transactional leadership: A two-sample study. J Manag. 1999;25:897-933.
404. Pillai R, Williams ES, Tan JJ. Are the scales tipped in favor of procedural or distributive justice? An investigation of the U.S., India, Germany, and Hong Kong (China). Int J Confl Manag. 2001;12:312-32.
405. Piotrkowski CS. Gender harassment, job satisfaction, and distress among employed white and minority women. J Occup Health Psychol. 1998;3:33-43.
406. Poon JML. Distributive justice, procedural justice, affective commitment, and turnover intention: A mediation–moderation framework. J Appl Soc Psychol. 2012;42:1505-32.
407. Porath CL, Pearson CM. Emotional and behavioral responses to workplace incivility and the impact of hierarchical status. J Appl Soc Psychol. 2012;42:E326-57.
408. Priesemuth M, Schminke M, Ambrose ML, Folger R. Abusive supervision climate: A multiple-mediation model of its impact on group outcomes. Acad Manag J. 2014;57:1513-34.
409. Pseekos AC, Bullock-Yowell E, Dahlen ER. Examining Holland’s person–environment fit, workplace aggression, interpersonal conflict, and job satisfaction. J Employ Couns. 2011;48:63-71.
410. Quine, L. Workplace bullying in nurses. J Health Psychol. 2001;6:73-84.
411. Rafferty AE, Restubog SLD, Jimmieson NL. Losing sleep: Examining the cascading effects of supervisors’ experience of injustice on subordinates’ psychological health. Work Stress. 2010;24:36-55.
412. Ragins BR, Scandura TA. Antecedents and work-related correlates of reported sexual harassment: An empirical investigation of competing hypotheses. Sex Roles. 1995;32:429-55.
413. Rainey DW. Stress, burnout, and intention to terminate among umpires. J Sport Behav. 1995;18:312-23.
414. Rainey DW. Sources of stress, burnout, and intention to terminate among basketball referees. J Sport Behav. 1999;22:578-90.
415. Ramamoorthy N, Flood PC, Slattery T, Sardessai R. Determinants of innovative work behaviour: Development and test of an integrated model. Creat Innov Manag. 2005;14:142-50.
416. Ramaswami SN, Singh J. Antecedents and consequences of merit pay fairness for industrial salespeople. J Mark. 2003;67:46-66.
417. Raver JL, Nishii LH. Once, twice, or three times as harmful? Ethnic harassment, gender harassment, and generalized workplace harassment. J Appl Psychol. 2010;95:236-54.
418. Read E, Laschinger HK. Correlates of new graduate nurses’ experiences of workplace mistreatment. J Nurs Adm. 2013;43:221-8.
419. Reio Jr TG. Supervisor and coworker incivility: Testing the Work Frustration-Aggression Model. Adv Dev Hum Resour. 2011;13:54-68.
420. Reknes I, Pallesen S, Magerøy N, Moen BE, Bjorvatn B, Einarsen S. Exposure to bullying behaviors as a predictor of mental health problems among Norwegian nurses: Results from the prospective SUSSH-survey. Int J Nurs Stud. 2014;51:479-87.
421. Renn R, Allen D, Huning T. The relationship of social exclusion at work with self-defeating behavior and turnover. J Soc Psychol. 2013;153:229-49.
422. Renn RW. Participation's effect on task performance: Mediating roles of goal acceptance and procedural justice. J Bus Res. 1998;41:115-25.
423. Restubog SLD, Bordia P, Bordia S. The interactive effects of procedural justice and equity sensitivity in predicting responses to psychological contract breach: An interactionist perspective. J Bus Psychol. 2009;24:165-78.
424. Restubog SLD, Scott KL, Zagenczyk TJ. When distress hits home: The role of contextual factors and psychological distress in predicting employees’ responses to abusive supervision. J Appl Psychol. 2011;96:713-29.
425. Richardsen AM, Burke RJ, Leiter MP. Occupational demands, psychological burnout and anxiety among hospital personnel in Norway. Anxiety Stress Coping. 1992;5:55-68.
426. Richman-Hirsch WL, Glomb TM. Are men affected by the sexual harassment of women? Effects of ambient sexual harassment on men. In: Brett JM, Drasgow F, editors. The Psychology of work: Theoretically based empirical research. Mahwah: Psychology Press; 2002. p. 121-40.
427. Rifai HA. A test of the relationships among perceptions of justice, job satisfaction, affective commitment and organizational citizenship behavior. Gadjah Mada Int J Bus. 2005;7:131-54.
428. Robbins TL, Summers TP, Miller JL. Intra- and inter-justice relationships: Assessing the direction. Hum Relat. 2000;53:1329-55.
429. Rodríguez-Muñoz A, Baillien E, De Witte H, Moreno-Jiménez B, Pastor JC. Cross-lagged relationships between workplace bullying, job satisfaction and engagement: Two longitudinal studies. Work Stress. 2009;23:225-43.
430. Rodríguez-Muñoz A, Notelaers G, Moreno-Jiménez B. Workplace bullying and sleep quality: The mediating role of worry and need for recovery. Behav Psychol. 2011;19:453-68.
431. Rodwell J, Noblet A, Demir D, Steane P. The impact of the work conditions of allied health professionals on satisfaction, commitment and psychological distress. Health Care Manag Rev. 2009;34:273-83.
432. Rogers K-A, Kelloway EK. Violence at work: Personal and organizational outcomes. J Occup Health Psychol. 1997;2:63-71.
433. Rösler U, Gebele N, Hoffmann K, Morling K, Müller A, Rau R, Stephan U. Cortisol – ein geeigneter physiologischer Indikator für Belastungen am Arbeitsplatz? [Cortisol – a useful physiological parameter for work-related stress?]. Z Arb Organ. 2010;54:68-82.
434. Rospenda KM, Richman JA, Shannon CA. Patterns of workplace harassment, gender, and use of services: An update. J Occup Health Psychol. 2006;11:379-93.
435. Sá L, Fleming M. Bullying, burnout, and mental health amongst Portuguese nurses. Issues Ment Health Nurs. 2008;29:411-26.
436. Sager JK. A longitudinal assessment of change in sales force turnover. J Acad Mark Sci. 1991;19:25-36.
437. Saks AM. Antecedents and consequences of employee engagement. J Manag Psychol. 2006;21:600-19.
438. Sakurai K, Jex SM. Coworker incivility and incivility targets’ work effort and counterproductive work behaviors: The moderating role of supervisor social support. J Occup Health Psychol. 2012;17:150-61.
439. Santos A, Leather P, Dunn J, Zarola A. Gender differences in exposure to co-worker and public-initiated violence: Assessing the impact of work-related violence and aggression in police work. Work Stress. 2009;23:137-54.
440. Savelsbergh C, Gevers JMP, van der Heijden BIJM, Poell RF. Team role stress: Relationships with team learning and performance in project teams. Group Organ Manag. 2012;37:67-100.
441. Schappe SP. The influence of job satisfaction, organizational commitment, and fairness perceptions on organizational citizenship behavior. J Psychol. 1998;132:277-90.
442. Schat ACH, Frone MR. Exposure to psychological aggression at work and job performance: The mediating role of job attitudes and personal health. Work Stress. 2011;25:23-40.
443. Schat ACH, Kelloway EK. Effects of perceived control on the outcomes of workplace aggression and violence. J Occup Health Psychol. 2000;5:386-402.
444. Schat ACH, Kelloway EK. Reducing the adverse consequences of workplace aggression and violence: The buffering effects of organizational support. J Occup Health Psychol. 2003;8:110-22.
445. Schaubroeck J, Fink LS. Facilitating and inhibiting effects of job control and social support on stress outcomes and role behavior: A contingency model. J Organ Behav. 1998;19:167-95.
446. Schaubroeck J, Walumbwa FO, Ganster DC, Kepes S. Destructive leader traits and the neutralizing influence of an “enriched“ job. Leadersh Q. 2007;18:236-51.
447. Schaufeli WB, Maassen GH, Bakker AB, Sixma HJ. Stability and change in burnout: A 10-year follow-up study among primary care physicians. J Occup Organ Psychol. 2011;84:248-67.
448. Schilpzand MC, Martins LL, Kirkman BL, Lowe KB, Chen ZX. The relationship between organizational justice and organizational citizenship behaviour: The role of cultural value orientations. Manag Organ Rev. 2013;9:345-74.
449. Schneider B, Snyder RA. Some relationships between job satisfaction and organizational climate. J Appl Psychol. 1975;60:318-28.
450. Schneider KT, Hitlan RT, Radhakrishnan P. An examination of the nature and correlates of ethnic harassment experiences in multiple contexts. J Appl Psychol. 2000;85:3-12.
451. Schneider KT, Swan S, Fitzgerald LF. Job-related and psychological effects of sexual harassment in the workplace: Empirical evidence from two organizations. J Appl Psychol. 1997;82:401-15.
452. Semmer NK, Tschan F, Meier LL, Facchin S, Jacobshagen N. Illegitimate tasks and counterproductive work behavior. Appl Psychol-Int Rev. 2010;59:70-96.
453. Seppälä T, Lipponen J, Pirttilä-Backman A-M, Lipsanen J. A trust-focused model of leaders’ fairness enactment. J Pers Psychol. 2012;11:20-30.
454. Settles IH, Cortina LM, Malley J, Stewart AJ. The climate for women in academic science: The good, the bad, and the changeable. Psychol Women Q. 2006;30:47-58.
455. Shaffer MA, Joplin JRW, Bell MP, Lau T, Oguz C. Gender discrimination and job-related outcomes: A cross-cultural comparison of working women in the United States and China. J Vocat Behav. 2000;57:395-427.
456. Shahzad A, Siddiqui MA, Zakaria M. Linking organizational justice with organization citizenship behaviors: Collectivism as moderator. Pak J Commer Soc Sci. 2014;8:900-13.
457. Shao P, Resick CJ, Hargis MB. Helping and harming others in the workplace: The roles of personal values and abusive supervision. Hum Relat. 2011;64:1051-78.
458. Sharoni G, Tziner A, Fein EC, Shultz T, Shaul K, Zilberman L. Organizational citizenship behavior and turnover intentions: Do organizational culture and justice moderate their relationship? J Appl Soc Psychol. 2012;42:E267-94.
459. Shim J, Chang HJ. The relationship between workplace incivility and the intention to leave; implication to HRD. In: Candel Torres I, Gómez Chova L, López Martínez A, editors. ICERI2011: 4th International Conference of Education, Research and Innovation: Conference Proceedings. International Association of Technology, Education and Development (IATED); 2011. p. 674-9.
460. Shoss MK, Eisenberger R, Restubog SLD, Zagenczyk TJ. Blaming the organization for abusive supervision: The roles of perceived organizational support and supervisor’s organizational embodiment. J Appl Psychol. 2013;98:158-68.
461. Siers B. Relationships among organisational justice perceptions, adjustment, and turnover of United States-based expatriates. Appl Psychol-Int Rev. 2007;56:437-59.
462. Simons T, Roberson Q. Why managers should care about fairness: The effects of aggregate justice perceptions on organizational outcomes. J Appl Psychol. 2003;88:432-43.
463. Sinclair RR, Martin JE, Croll LW. A threat-appraisal perspective on employees’ fears about antisocial workplace behavior. J Occup Health Psychol. 2002;7:37-56.
464. Singh AK, Jayaratne S, Siefert K, Chess WA. Emotional support and social undermining as predictors of well-being. Indian J Soc Work. 1995;56:349-59.
465. Skogstad A, Einarsen S, Torsheim T, Aasland MS, Hetland H. The destructiveness of laissez-faire leadership behavior. J Occup Health Psychol. 2007;12:80-92.
466. Slåtten T, Svensson G, Sværi S. Service quality and turnover intentions as perceived by employees: Antecedents and consequences. Pers Review. 2011;40:205-21.
467. Slattery JP, Selvarajan TT, Anderson JE. The influences of new employee development practices upon role stressors and work-related attitudes of temporary employees. Int J Hum Resour Manag. 2008;19:2268-93.
468. Sliter KA, Sinclair R, Cheung J, McFadden A. Initial evidence for the buffering effect of physical activity on the relationship between workplace stressors and individual outcomes. Int J Stress Manag. 2014;21:348-60.
469. Sliter KA, Sliter MT, Withrow SA, Jex SM. Employee adiposity and incivility: Establishing a link and identifying demographic moderators and negative consequences. J Occup Health Psychol. 2012;17:409-24.
470. Sliter M, Jex S, Wolford K, McInnerney J. How rude! Emotional labor as a mediator between customer incivility and employee outcomes. J Occup Health Psychol. 2010;15:468-81.
471. Sliter M, Sliter K, Jex S. The employee as a punching bag: The effect of multiple sources of incivility on employee withdrawal behavior and sales performance. J Organ Behav. 2012;33:121-39.
472. Sloan MM. Unfair treatment in the workplace and worker well-being: The role of coworker support in a service work environment. Work Occup. 2012;39:3-34.
473. Smith LGE, Amiot CE, Callan VJ, Terry DJ, Smith JR. Getting new staff to stay: The mediating role of organizational identification. Br J Manag. 2012;23:45-64.
474. Sobiraj S, Korek S, Weseler D, Mohr G. When male norms don’t fit: Do traditional attitudes of female colleagues challenge men in non-traditional occupations? Sex Roles. 2011;65:798-812.
475. Sommer KL, Kulkarni M. Does constructive performance feedback improve citizenship intentions and job satisfaction? The roles of perceived opportunities for advancement, respect, and mood. Hum Resour Dev Q. 2012;23:177-201.
476. Song G, Liu H. Customer-related social stressors and emotional exhaustion: The mediating role of surface and deep acting. Soc Behav Pers. 2010;38:1359-66.
477. Sonnentag S, Unger D, Nägel IJ. Workplace conflict and employee well-being: The moderating role of detachment from work during off-job time. Int J Confl Manag. 2013;24:166-83.
478. Spector PE. Interactive effects of perceived control and job stressors on affective reactions and health outcomes for clerical workers. Work Stress. 1987;1:155-62.
479. Spector PE, Coulter ML, Stockwell HG, Matz MW. Perceived violence climate: A new construct and its relationship to workplace physical violence and verbal aggression, and their potential consequences. Work Stress. 2007;21:117-30.
480. Spector PE, Dwyer DJ, Jex SM. Relation of job stressors to affective, health, and performance outcomes: A comparison of multiple data sources. J Appl Psychol. 1988;73:11-9.
481. Spector PE, Fox S, Penney LM, Bruursema K, Goh A, Kessler S. The dimensionality of counterproductivity: Are all counterproductive behaviors created equal? J Vocat Behav. 2006;68:446-60.
482. Spector PE, O’Connell BJ. The contribution of personality traits, negative affectivity, locus of control and Type A to the subsequent reports of job stressors and job strains. J Occup Organ Psychol. 1994;67:1-11.
483. Spector PE, Zhou ZE. The moderating role of gender in relationships of stressors and personality with counterproductive work behavior. J Bus Psychol. 2014;29:669-81.
484. Spector PE, Zhou ZE, Yang L. (2010, October 30 - November 3). A longitudinal study relating workplace abuse to physical and psychological strains. Paper presented at the 11th Annual Meeting of the Southern Management Association, Ft. Lauderdale, FL, USA.
485. Spell CS, Arnold TJ. A multi-level analysis of organizational justice climate, structure, and employee mental health. J Manag. 2007;33:724-51.
486. Sprigg CA, Armitage CJ, Hollis K. Verbal abuse in the National Health Service: Impressions of the prevalence, perceived reasons for and relationships with staff psychological well-being. Emerg Med J. 2007;24:281-2.
487. Sprung JM, Jex SM. Work locus of control as a moderator of the relationship between work stressors and counterproductive work behavior. Int J Stress Manag. 2012;19:272-91.
488. Sprung JM, Sliter MT, Jex SM. Spirituality as a moderator of the relationship between workplace aggression and employee outcomes. Pers Individ Differ. 2012;53:930-34.
489. Stamper CL, Johlke MC. The impact of perceived organizational support on the relationship between boundary spanner role stress and work outcomes. J Manag. 2003;29:569-88.
490. Stedham Y, Mitchell MC. Sexual harassment in casinos: Effects on employee attitudes and behaviors. J Gambl Stud. 1998;14:381-400.
491. Stoetzer U, Ahlberg G, Johansson G, Bergman P, Hallsten L, Forsell Y, Lundberg I. Problematic interpersonal relationships at work and depression: A Swedish prospective cohort study. J Occup Health. 2009;51:144-51.
492. Streicher B, Jonas E, Maier GW, Frey D, Woschée R, Waßmer B. Test of the construct and criteria validity of a German measure of organizational justice. Eur J Psychol Assess. 2008;24:131-9.
493. Sulea C, Virga D, Maricutoiu LP, Schaufeli W, Zaborila Dumitru C, Sava FA. Work engagement as mediator between job characteristics and positive and negative extra-role behaviors. Career Dev Int. 2012;17:188-207.
494. Suliman AMT. Links between justice, satisfaction and performance in the workplace: A survey in the UAE and Arabic context. J Manag Dev. 2007;26:294-311.
495. Sutton RI. Job stress among primary and secondary schoolteachers: Its relationship to ill-being. Work Occup. 1984;11:7-28.
496. Taddei S, Contena B. Burnout in call centre workers between demands and resources. Boll Psicol Appl. 2010;261-262:37-48.
497. Taddei S, Vanni D. Customer-related social stress and burnout. A contribution to the Italian adaptation of the Customer-Related Social Stress Scale. Boll Psicol Appl. 2008;256:41-53.
498. Tao L, Weimei Y, Yi H. Study on the correlation of civil servant organization justice and turnover tendency based on the external job opportunities as mediating variable. In: Proceedings of the 2012 International Conference on Public Management (ICPM-2012). Atlantis Press; 2012. p. 64-9.
499. Taris TW, Peeters MCW, Le Blanc PM, Schreurs PJG, Schaufeli WB. From inequity to burnout: The role of job stress. J Occup Health Psychol. 2001;6:303-23.
500. Taylor SG, Kluemper DH. Linking perceptions of role stress and incivility to workplace aggression: The moderating role of personality. J Occup Health Psychol. 2012;17:316-29.
501. Tejeda MJ. Nondiscrimination policies and sexual identity disclosure: Do they make a difference in employee outcomes? Empl Responsib Rights J. 2006;18:45-59.
502. Tekleab AG, Bartol KM, Liu W. Is it pay levels or pay raises that matter to fairness and turnover? J Organ Behav. 2005;26:899-921.
503. Tepper BJ, Carr JC, Breaux DM, Geider S, Hu C, Hua W. Abusive supervision, intentions to quit, and employees’ workplace deviance: A power/dependence analysis. Organ Behav Hum Decis Process. 2009;109:156-67.
504. Tepper BJ, Duffy MK, Hoobler J, Ensley MD. Moderators of the relationships between coworkers’ organizational citizenship behavior and fellow employees’ attitudes. J Appl Psychol. 2004;89:455-65.
505. Tepper BJ, Henle CA, Lambert LS, Giacalone RA, Duffy MK. Abusive supervision and subordinates’ organization deviance. J Appl Psychol. 2008;93:721-32.
506. Tepper BJ, Lockhart D, Hoobler J. Justice, citizenship, and role definition effects. J Appl Psychol. 2001;86:789-96.
507. Tepper BJ, Moss SE, Duffy MK. Predictors of abusive supervision: Supervisor perceptions of deep-level dissimilarity, relationship conflict, and subordinate performance. Acad Manag J. 2011;54:279-94.
508. Thau S, Mitchell MS. Self-gain or self-regulation impairment? Tests of competing explanations of the supervisor abuse and employee deviance relationship through perceptions of distributive justice. J Appl Psychol. 2010;95:1009-31.
509. Thompson RC, Bailey LL, Joseph KM, Worley JA, Williams CA. Organizational change: Effects of fairness perceptions on cynicism (final report). Washington: Office of Aviation Medicine; 1999. p. 1-9.
510. Thoroughgood CN, Tate BW, Sawyer KB, Jacobs R. Bad to the bone: Empirically defining and measuring destructive leader behavior. J Leadersh Organ Stud. 2012;19:230-55.
511. Tsouloupas CN, Carson RL, Matthews R, Grawitch MJ, Barber LK. Exploring the association between teachers’ perceived student misbehaviour and emotional exhaustion: The importance of teacher efficacy beliefs and emotion regulation. Educ Psychol. 2010;30:173-89.
512. Tuckey MR, Neall AM. Workplace bullying erodes job and personal resources: Between- and within-person perspectives. J Occup Health Psychol. 2014;19:413-24.
513. Van der Doef M, Maes S. Teacher-specific quality of work versus general quality of work assessment: A comparison of their validity regarding burnout, (psycho)somatic well-being and job satisfaction. Anxiety Stress Coping. 2002;15:327-44.
514. Van Dick R, Wagner U. Stress and strain in teaching: A structural equation approach. Br J of Educ Psychol. 2001;71:243-59.
515. Van Dierendonck D, Schaufeli WB, Sixma HJ. Burnout among general practitioners: A perspective from Equity Theory. J Soc Clin Psychol. 1994;13:86-100.
516. Van Jaarsveld DD, Walker DD, Skarlicki DP. The role of job demands and emotional exhaustion in the relationship between customer and employee incivility. J Manag. 2010;36:1486-504.
517. Van Katwyk PT, Fox S, Spector PE, Kelloway EK. Using the Job-Related Affective Well-Being Scale (JAWS) to investigate affective responses to work stressors. J Occup Health Psychol. 2000;5:219-30.
518. Van Schalkwyk L-M, Els C, Rothmann Jr I. The moderating role of perceived organisational support in the relationship between workplace bullying and turnover intention across sectors in South Africa. SA J Hum Resour Manag. 2011;9:a384.
519. Vandenberghe C, Panaccio A, Bentein K, Mignonac K, Roussel P. Assessing longitudinal change of and dynamic relationships among role stressors, job attitudes, turnover intention, and well‐being in neophyte newcomers. J Organ Behav. 2011;32:652-71.
520. Vartia MA-L. Consequences of workplace bullying with respect to the well-being of its targets and the observers of bullying. Scand J Work Environ Health. 2001;27:63-9.
521. Vie TL, Glasø L, Einarsen S. Does trait anger, trait anxiety or organisational position moderate the relationship between exposure to negative acts and self-labelling as a victim of workplace bullying? Nord Psychol. 2010;62:67-79.
522. Vie TL, Glasø L, Einarsen S. Health outcomes and self-labeling as a victim of workplace bullying. J Psychosom Res. 2011;70:37-43.
523. Volmer J, Binnewies C, Sonnentag S, Niessen C. Do social conflicts with customers at work encroach upon our private lives? A diary study. J Occup Health Psychol. 2012;17:304-15.
524. Von Hippel C, Issa M, Ma R, Stokes A. Stereotype threat: Antecedents and consequences for working women. Eur J Soc Psychol. 2011;41:151-61.
525. Walsh BM, Hitlan RT. Organizational stress: Investigating the impact of dual harassment experiences on appraisal and outcomes. N Am J Psychol. 2007;9:331-46.
526. Walsh G. Unfriendly customers as a social stressor – An indirect antecedent of service employees’ quitting intention. Eur Manag J. 2011;29:67-78.
527. Wang M, Liao H, Zhan Y, Shi J. Daily customer mistreatment and employee sabotage against customers: Examining emotion and resource perspectives. Acad Manag J. 2011;54:312-34.
528. Wang M, Liu S, Liao H, Gong Y, Kammeyer-Mueller J, Shi J. Can’t get it out of my mind: Employee rumination after customer mistreatment and negative mood in the next morning. J Appl Psychol. 2013;98:989-1004.
529. Wang W, Mao J, Wu W, Liu J. Abusive supervision and workplace deviance: The mediating role of interactional justice and the moderating role of power distance. Asia Pac J Hum Resour. 2012;50:43-60.
530. Waraich SB, Bhardwaj G. Perception of workforce reduction scenario and coping strategies of survivors: An empirical study. South Asian J Manag. 2012;19: 34-49.
531. Wegge J, Van Dick R, von Bernstorff C. Emotional dissonance in call centre work. J Manag Psychol. 2010;25:596-619.
532. Wei F, Si S. Tit for tat? Abusive supervision and counterproductive work behaviors: The moderating effects of locus of control and perceived mobility. Asia Pac J Manag. 2013;30:281-96.
533. Weiss V. Zufriedenheit und Wohlbefinden verbleibender MitarbeiterInnen nach Personalabbau [Satisfaction and well-being of remaining employees after personnel reduction]. Wirtschaftspsychol. 2005;7:81-92.
534. Wesolowski MA, Mossholder KW. Relational demography in supervisor–subordinate dyads: Impact on subordinate job satisfaction, burnout, and perceived procedural justice. J Organ Behav. 1997;18:351-62.
535. Wheeler AR, Halbesleben JRB, Whitman MV. The interactive effects of abusive supervision and entitlement on emotional exhaustion and co‐worker abuse. J Occup Organ Psychol. 2013;86:477-96.
536. Williams S, Pitre R, Zainuba M. Justice and organizational citizenship behavior intentions: Fair rewards versus fair treatment. J Soc Psychol. 2002;142:33-44.
537. Willingstorfer B, Schaper N, Sonntag K. Mobbingmasse und -faktoren sowie bestehende Zusammenhänge mit sozialen Arbeitsplatzbedingungen [Relationships between measures of workplace bullying and social conditions at the workplace]. Z Arb Organ. 2002;46:111-25.
538. Winstanley S, Whittington R. Anxiety, burnout and coping styles in general hospital staff exposed to workplace aggression: A cyclical model of burnout and vulnerability to aggression. Work Stress. 2002;16:302-15.
539. Wong Y-T. Job security and justice: Predicting employees’ trust in Chinese international joint ventures. Int J Hum Resour Manag. 2012;23:4129-44.
540. Wong Y-T, Ngo H-Y, Wong C-S. Perceived organizational justice, trust, and OCB: A study of Chinese workers in joint ventures and state-owned enterprises. J World Bus. 2006;41:344-55.
541. Wong Y-T, Wong C-S, Ngo H-Y. The effects of trust in organisation and perceived organisational support on organisational citizenship behaviour: A test of three competing models. Int J Hum Resour Manag. 2012;23:278-93.
542. Workman M. A field study of corporate employee monitoring: Attitudes, absenteeism, and the moderating influences of procedural justice perceptions. Inf Organ. 2009;19:218-32.
543. Wu M, Huang X, Li C, Liu W. Perceived interactional justice and trust‐in‐supervisor as mediators for paternalistic leadership. Manag Organ Rev. 2012;8:97-121.
544. Wu T-Y, Hu C. Abusive supervision and employee emotional exhaustion: Dispositional antecedents and boundaries. Group Organ Manag. 2009;34:143-69.
545. Xianyu Y, Lambert VA. Investigation of the relationships among workplace stressors, ways of coping, and the mental health of Chinese nurses. Nurs Health Sci. 2006;8:147-55.
546. Xu E, Huang X, Lam CK, Miao Q. Abusive supervision and work behaviors: The mediating role of LMX. J Organ Behav. 2012;33:531-43.
547. Yang J, Diefendorff JM. The relations of daily counterproductive workplace behavior with emotions, situational antecedents, and personality moderators: A diary study in Hong Kong. Pers Psychol. 2009;62:259-95.
548. Ybema JF, van den Bos K. Effects of organizational justice on depressive symptoms and sickness absence: A longitudinal perspective. Soc Sci Med. 2010;70:1609-17.
549. Zapf D, Knorz C, Kulla M. On the relationship between mobbing factors, and job content, social work environment, and health outcomes. Eur J Work Organ Psychol. 1996;5:215-37.
550. Zapf D, Seifert C, Schmutte B, Mertini H, Holz M. Emotion work and job stressors and their effects on burnout. Psychol Health. 2001;16:527-45.
551. Zhang G, Lee G, Zou X. The mediating role of procedural justice between participation in decision-making and organizational citizenship behavior: An empirical study about Skeleton Government Civilian in China. Psychol. 2010;1:300-4.
552. Zhang H, Agarwal NC. The mediating roles of organizational justice on the relationships between HR practices and workplace outcomes: An investigation in China. Int J Hum Resour Manag. 2009;20:676-93.
553. Zhang J. A study on the relationship between management team conflict and organizational citizenship behavior in colleges and universities: The mediating effect of organizational justice. In: Lan H, Yang Y-H, editors. 2013 International Conference on Management Science and Engineering: 20th Annual Conference Proceedings. Piscataway: Institute of Electrical and Electronic Engineers; 2013. p. 1427-32.
554. Zhang L, Morand D. The linkage between status-leveling symbols and work attitudes. J Manag Psychol. 2014;29:973-93.
555. Zhao H, Peng Z, Chen H-K. Compulsory citizenship behavior and organizational citizenship behavior: The role of organizational identification and perceived interactional justice. J Psychol. 2014;148:177-96.
556. Zhao H, Peng Z, Sheard G. Workplace ostracism and hospitality employees’ counterproductive work behaviors: The joint moderating effects of proactive personality and political skill. Int J Hosp Manag. 2013;33:219-27.
557. Zickar MJ, Balzer WK, Aziz S, Wryobeck JM. The moderating role of social support between role stressors and job attitudes among Roman Catholic priests. J Appl Soc Psychol.
